# Metagenome-assembled genomes reveal microbial signatures and metabolic pathways linked to coronary artery disease

**DOI:** 10.1128/msystems.00954-25

**Published:** 2025-11-06

**Authors:** Soomin Lee, Shahbaz Raza, Eun-Ju Lee, Yoosoo Chang, Seungho Ryu, Hyung-Lae Kim, Si-Hyuck Kang, Han-Na Kim

**Affiliations:** 1 Department of Clinical Research Design and Evaluation, Samsung Advanced Institute for Health Sciences & Technology, Sungkyunkwan University 198720, Seoul, Republic of Korea; 2 Center for Cohort Studies, Total Healthcare Center, Kangbuk Samsung Hospital, Sungkyunkwan University School of Medicine 198720, Seoul, Republic of Korea; 3 Department of Occupational and Environmental Medicine, Kangbuk Samsung Hospital, Sungkyunkwan University School of Medicine 198720, Seoul, Republic of Korea; 4 Department of Biochemistry, College of Medicine, Ewha Womans University 92203, Seoul, Republic of Korea; 5 Cardiovascular Center, Seoul National University Bundang Hospital, Seongnam-si, Republic of Korea; 6 Department of Internal Medicine, Seoul National University 26725, Seoul, Republic of Korea; 7 Center for Clinical Epidemiology, Samsung Medical Center, Sungkyunkwan University 198720, Seoul, Republic of Korea; University of Rhode Island, Kingston, Rhode Island, USA

**Keywords:** coronary artery disease, cardiovascular disease, gut microbiome, shotgun metagenomics, metagenome-assembled genome

## Abstract

Gut microbiota has emerged as a critical factor influencing cardiovascular disease (CVD) risk, particularly coronary artery disease (CAD) development. Using fecal metagenomic shotgun sequencing, we investigated gut microbiota signatures associated with CAD and provided strain-resolved insights through metagenome-assembled genome (MAG) reconstruction. We analyzed 14 patients with CAD and 28 propensity score-matched healthy controls. Differential abundance analysis identified 15 CAD-associated bacterial species. Members of the *Lachnospiraceae* family, previously associated with trimethylamine-N-oxide production, were significantly enriched in patients with CAD. Conversely, short-chain fatty acid-producing bacteria *Slackia isoflavoniconvertens* and *Faecalibacterium prausnitzii* were depleted, suggesting a potential contribution to gut-mediated inflammation and metabolic dysregulation. Metabolic pathway analysis revealed significant urea cycle and L-citrulline biosynthesis enrichment in CAD cases, with *Alistipes* and *Coprococcus* as key contributors. Among predicted metabolites, inosine, which is implicated in coronary artery relaxation, was elevated in patients with CAD, whereas C18:0e MAG and α-muricholate were depleted. A random forest model achieved a mean AUC of 0.89 for CAD classification, with improved performance when integrating microbial taxa and metabolites. CAD-derived MAGs showed metabolic signatures linked to inflammatory dysbiosis and cardiovascular dysfunction, such as enriched N_2_ fixation and sulfite reduction. Strain-resolved comparative genomic analysis of MAGs revealed distinctive functional characteristics between CAD-derived and control-derived strains of *Akkermansia muciniphila* and *Megamonas fumiformis. F. prausnitzii* MAG from the control group carried non-trimethylamine-producing gene, *mtxB*, suggesting its potential protective role in CAD pathophysiology. These findings provide insights into gut microbial alterations in CAD and highlight potential targets for microbiome-based therapeutic interventions to reduce CVD risk.

**IMPORTANCE:**

Gut microbiota plays a pivotal role in cardiovascular disease; however, its specific contribution to coronary artery disease (CAD) remains underexplored. This study identified distinct microbial signatures associated with CAD, including the enrichment of pro-inflammatory bacterial taxa and depletion of short-chain fatty acid-producing bacteria, which may contribute to systemic inflammation and metabolic dysregulation. Perturbations in key pathways, such as the urea cycle and glycolysis, suggest metabolic links between the gut microbiota and CAD. Additionally, the metagenome-assembled genome-based analysis revealed strain-resolved functional heterogeneity that shapes host-microbe interactions and may contribute to CAD pathophysiology. These findings provide novel insights into gut dysbiosis in CAD and highlight the potential of microbiome-targeted therapeutic strategies in precision medicine.

## INTRODUCTION

Cardiovascular disease (CVD) is a leading cause of global mortality and morbidity, with coronary artery disease (CAD) representing a prevalent form of CVD characterized by myocardial dysfunction and lesions due to coronary artery stenosis and inadequate blood supply (1). Approximately half of the adults in the USA suffer from CVD, for ~25% of which it is fatal (1). In South Korea, CVD is the second leading cause of death among adults after cancer (2). Although genetic and environmental factors contribute to CAD, emerging research highlights the pivotal role of the gut microbiota in disease progression via its impact on systemic inflammation, lipid metabolism, and atherosclerosis (3). However, the specific microbial taxa and functional pathways involved remain poorly understood and require further investigation into their mechanistic roles.

Gut microbiota-derived metabolites, particularly trimethylamine-N-oxide (TMAO), have been implicated in CAD pathogenesis by linking dietary choline metabolism to systemic inflammation, endothelial dysfunction, and progression (4). The microbial conversion of dietary choline and L-carnitine into trimethylamine (TMA), followed by hepatic oxidation to TMAO, promotes vascular inflammation, platelet hyperreactivity, and foam cell formation, contributing to plaque development and cardiovascular risk (3, 5). In addition to TMAO, the gut microbiota modulates lipid metabolism, bile acid transformation, and short-chain fatty acid (SCFA) production, influencing host metabolism and immune responses relevant to CAD (6).

Emerging evidence suggests that microbiota-based interventions may help to modulate cardiovascular risk. A randomized controlled trial demonstrated that probiotic supplementation with *Lactobacillus rhamnosus* significantly reduced systemic inflammation, metabolic endotoxemia, and inflammatory cytokine levels in patients (7). Similarly, dietary interventions targeting gut microbiota modifications have shown potential in improving lipid metabolism and vascular function, emphasizing the therapeutic relevance of microbiome modulation in cardiovascular health (8).

Despite these insights, CAD-associated microbiota remained incompletely characterized, especially at the species and strain levels. Although 16S rRNA sequencing has revealed broad compositional shifts, it lacks species- and strain-level functional insights (9). In contrast, shotgun metagenomics enables species-level classification and metabolic pathway reconstruction (10), providing a more comprehensive view of microbial contributions to CAD. A key advancement is the reconstruction of metagenome-assembled genomes (MAGs), which allows *de novo* assembly of microbial genomes, including those of uncultured and low-abundance microbes. Thus, they offer deep insights into microbial diversity and functional capabilities (11).

In this study, we used shotgun metagenomics to comprehensively profile the gut microbiomes of patients with CAD and healthy controls by examining both taxonomic and functional alterations. Our objectives were to (i) identify differences in microbial composition between patients with CAD and healthy individuals, (ii) characterize metabolic pathways and predict gut metabolites linked to CAD, and (iii) reconstruct MAGs to uncover uncharacterized genomes associated with CAD. By emphasizing strain-level taxonomy and metabolic functions, this study extends beyond traditional taxonomic profiling to explore how microbial functions and metabolic interactions contribute to the pathophysiology of CAD.

## RESULTS

### Basic characteristics of participants

After propensity score matching (PSM), 42 participants were included in the final analysis, comprising 14 patients with CAD and 28 matched controls (Table 1). The mean age of participants was 53.3 years (range: 40–70 years), and the mean body mass index (BMI) was 24.5 (range: 19.0–30.7). Three of the participants were female. Before PSM, age and high-sensitivity C-reactive protein levels differed significantly between the groups (*t*-test, *P* < 0.05; Table S1, Fig. S1); however, these differences were no longer significant after PSM, confirming the balance achieved through the matching process (Table 1). Shotgun metagenomic sequencing yielded an average of 45,844,546 raw reads per participant, of which 39,651,636 remained after host DNA was removed and were used for further analyses (Table S2).

**TABLE 1:** Basic characteristics of all the participants after propensity score matching^*a*^

Characteristic	Overall (*n* = 42)	Case (*n* = 14)	Control (*n* = 28)	*P-*value
Sex (%)				
Female	3	1 (7.2%)	2 (7.1%)	0.579
Male	39	13 (92.8%)	26 (92.9%)	
Age (years), mean ± SD	53.3 ± 9.2	52.4 ± 9.5	53.7 ± 9.2	0.68
BMI (kg/m), mean ± SD	24.7 ± 3.0	24.7 ± 3.0	24.4 ± 3.0	0.729
TG (mg/dL), mean ± SD	128.4 ± 53.7	109.5 ± 27.6	136.9 ± 60.5	0.051
HDL-C (mg/dL), mean ± SD	54.3 ± 12.5	51.5 ± 12.4	55.6 ± 12.6	0.326
Glucose (mg/dL), mean ± SD	96.8 ± 9.5	97.2 ± 11.1	96.6 ± 8.9	0.842
SBP (mmHg), mean ± SD	112.8 ± 9.6	110.8 ± 9.7	113.6 ± 9.7	0.395
DBP (mmHg), mean ± SD	74.0 ± 7.2	72.7 ± 7.0	74.6 ± 7.4	0.439
HCRP (mg/L)	0.05 ± 0.04	0.04 ± 0.02	0.05 ± 0.04	0.317
CRP (mg/L)	0.05 ± 0.05	0.05 ± 0.04	0.06 ± 0.05	0.712

^*a*^ BMI, body mass index; TG, triglyceride; HDL-C, high-density lipoprotein cholesterol; SBP, systolic blood pressure; DBP, diastolic blood pressure; HCRP, high-sensitivity C-reactive protein; CRP, C-reactive protein.

### Microbial community structure in patients with CAD

Among 42 participants, 520 bacterial species were identified. The most prevalent species across all individuals were *Prevotella copri* (recently renamed to *Segatella copri*, 14.37%), *Phocaeicola coprocola* (4.78%), and *Phocaeicola vulgatus* (4.44%; Table S3). Microbial diversity analysis revealed no significant differences in alpha diversity between patients with CAD and controls, as assessed using the Chao, Simpson, Shannon, and Pielou indices (Wilcoxon rank-sum test, *P* > 0.05; Fig. S2A). Similarly, Bray–Curtis distance-based beta diversity did not show significant differences between groups (permutational multivariate analysis of variance, PERMANOVA, *P* > 0.05; Fig. S2B).

Differential abundance analysis identified 15 bacterial species with significantly different relative abundances between patients with CAD and controls (linear regression model, *P* < 0.05). Among these, seven bacterial species, including *CAG-303 sp000437755*, *AM51-8 sp003478275*, *Ventrimonas sp900538475,* and *UBA644 sp900547165*, were significantly more abundant in the CAD cases (Table 2). In contrast, eight bacterial species, including *Slackia_A isoflavoniconvertens*, *Faecalibacterium prausnitzii*, *Catenibacterium sp000437715*, *Prevotella copri_A*, *Lachnospira eligens_A*, and *Holdemanella porci*, were significantly depleted in CAD cases compared with those in the control group. These findings suggest potential taxonomic shifts in the gut microbiome of patients with CAD, characterized by an increased abundance of certain bacterial taxa and depletion of SCFA-producing species.

**TABLE 2:** Differentially abundant bacterial species in the case and control groups^*a*^

Taxonomy	Coefficient	Relative abundance		*P-*value
		Case	Control	
p__Firmicutes_A;c__Clostridia;o__Lachnospirales;f__Lachnospiraceae;g__*CAG-303*;s__*CAG-303 sp000437755*	3.262	0.162	0.003	0.004
p__Actinobacteriota;c__Coriobacteriia;o__Coriobacteriales;f__Eggerthellaceae;g__*Slackia_A*;s__*Slackia_A isoflavoniconvertens*	−2.017	0.000	0.050	0.013
p__Firmicutes_A;c__Clostridia;o__Lachnospirales;f__Lachnospiraceae;g__*AM51-8*;s__*AM51-8 sp003478275*	2.724	0.340	0.055	0.015
p__Firmicutes_A;c__Clostridia;o__Oscillospirales;f__Ruminococcaceae;g__*Faecalibacterium*;s__*Faecalibacterium prausnitzii*	−2.473	0.616	1.744	0.024
p__Firmicutes;c__Bacilli;o__Erysipelotrichales;f__Coprobacillaceae;g__*Catenibacterium*;s__*Catenibacterium sp000437715*	−2.493	0.034	0.209	0.024
p__Firmicutes_A;c__Clostridia;o__Oscillospirales;f__Oscillospiraceae;g__CAG-83;s__*CAG-83 sp900757415*	−1.052	0	0.014	0.029
p__Firmicutes;c__Bacilli;o__Erysipelotrichales;f__Coprobacillaceae;g__*Faecalibacillus*;s__*Faecalibacillus intestinalis*	1.972	0.072	0.017	0.031
p__Firmicutes_A;c__Clostridia;o__Oscillospirales;f__Acutalibacteraceae;g__*UBA737*;s__*UBA737 sp002431945*	1.300	0.234	0.000	0.033
p__Bacteroidota;c__Bacteroidia;o__Bacteroidales;f__Bacteroidaceae;g__*Prevotella*;s__*Prevotella copri_A*	−3.708	3.34E−04	4.15E+00	0.035
p__Firmicutes_A;c__Clostridia;o__Lachnospirales;f__Lachnospiraceae;g__*Lachnospira*;s__*Lachnospira eligens_A*	−3.036	0.047	0.429	0.039
p__Firmicutes;c__Bacilli;o__Erysipelotrichales;f__Erysipelotrichaceae;g__*Holdemanella*;s__*Holdemanella porci*	−2.541	0.020	0.146	0.041
p__Firmicutes_A;c__Clostridia;o__Oscillospirales;f__Oscillospiraceae;g__*Lawsonibacter*;s__*Lawsonibacter sp014287875*	−1.199	0.002	0.022	0.041
p__Firmicutes_A;c__Clostridia;o__UBA1381;f__UBA1381;g__*CAG-41*;s__*CAG-41 sp900066215*	3.886	0.493	0.236	0.042
p__Firmicutes_A;c__Clostridia;o__Lachnospirales;f__Lachnospiraceae;g__*Ventrimonas*;s__*Ventrimonas sp900538475*	1.850	0.078	0.005	0.043
p__Firmicutes_A;c__Clostridia;o__Oscillospirales;f__UBA644;g__*UBA644*;s__*UBA644 sp900547165*	1.086	4.03E−03	3.75E−04	0.046

^*a*^ *P-*values were generated from the generalized linear model using Microbiome Multivariable Association with Linear Model 2 (MaAsLin2) between the two groups. Significantly different species were also observed (*P* < 0.05, linear regression model). The coefficients represent the direction of significance in each case. Negative coefficient values indicate higher abundance in the control group.

### Functional signature of gut microbiota in patients with CAD

**Fig 1:**
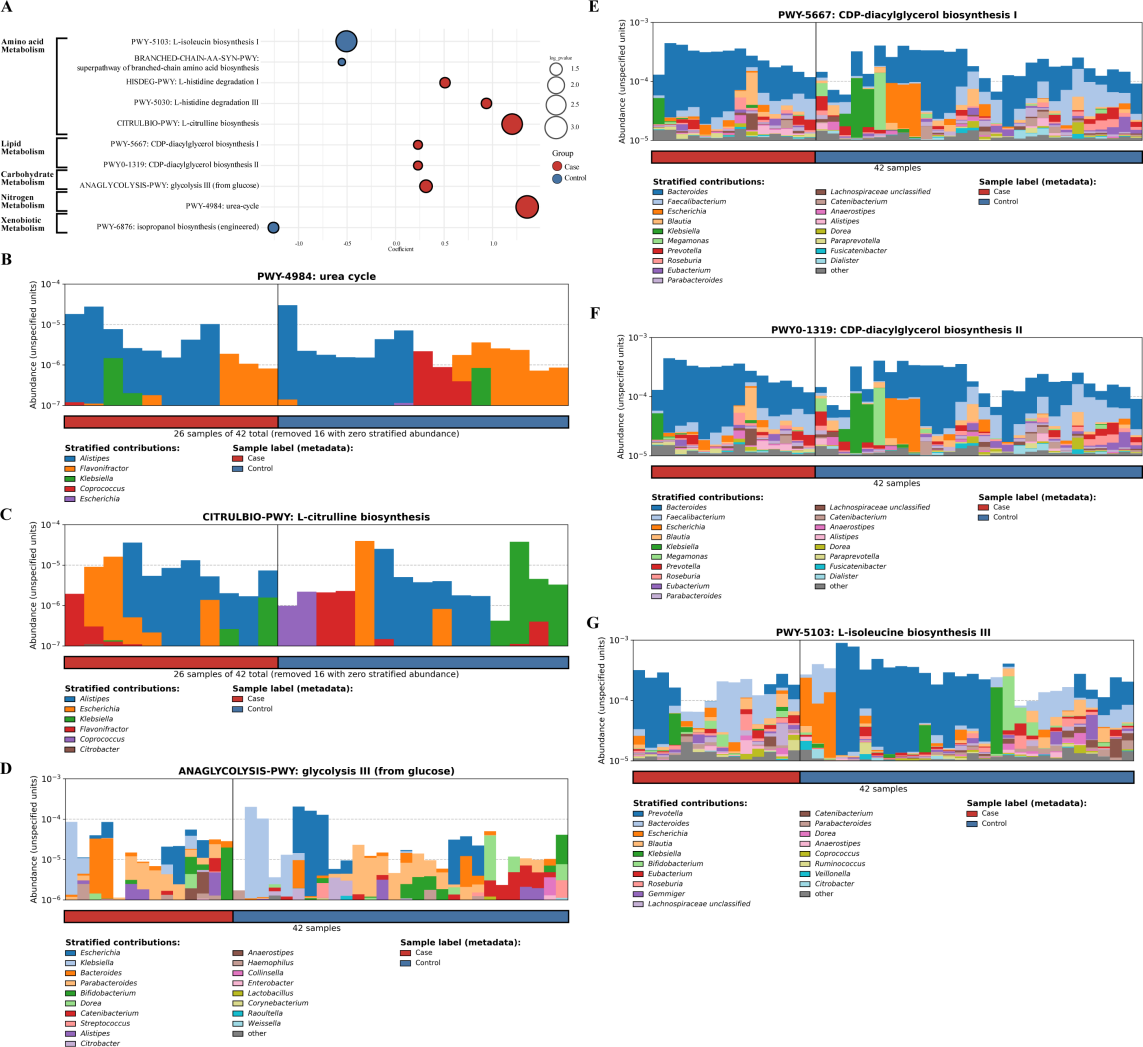
Metabolic pathways associated with coronary artery disease (CAD). (**A**) Bubble plot for differentially abundant pathways between case and control. *P*-values were calculated from differential abundance analysis with the Microbiome Multivariable Association with Linear Models 2 (MaAsLin2). The size of the bubble is based on *P*-values, where a larger size represents higher significance (lower *P*-value). The color of the bubble is based on its abundance in the case or control groups. Red color indicates higher abundance in cases, and blue represents higher abundance in the control group. (**B**) Contribution of bacterial genera in the abundance of the urea cycle pathway, (**C**) L-citrulline biosynthesis, (**D**) glycolysis III (from glucose), (**E**) CDP-diacylglycerol biosynthesis I, (**F**) CDP-diacylglycerol biosynthesis II, and (**G**) L-isoleucine biosynthesis III. The plot was generated with the HUMAnN3 bar graph plugin. We used the options of Bray–Curtis, and samples were sorted based on metadata.

Functional pathway analysis identified 10 MetaCyc metabolic pathways that differed significantly between patients with CAD and controls (linear regression model, *P* < 0.05; Fig. 1A). Among these, seven pathways, including the urea cycle, L-citrulline biosynthesis, glycolysis III (from glucose), and CDP-diacylglycerol biosynthesis, were significantly enriched in patients with CAD. Conversely, three metabolic pathways, including L-isoleucine biosynthesis III and the superpathway of branched-chain amino acid (BCAA) biosynthesis, were significantly depleted in patients with CAD.

To quantify the functional contributions of bacterial communities, we utilized the HUMAnN3 framework and stratified these functions based on the contributing species, referred to as “contributional diversity” of the function (12). In the urea cycle pathway, the predominant contributing genera were *Alistipes* and *Flavonifractor* (Fig. 1B). At the species level, *Alistipes finegoldii* and *Flavonifractor plautii* were the primary contributors, whereas *Alistipes onderdonkii* was detected exclusively in patients with CAD, and *Coprococcus catus* mainly contributed to controls (Fig. S3). In the L-citrulline biosynthesis pathway, *Alistipes*, *Escherichia,* and *Klebsiella* were the predominant contributors. *Escherichia* was enriched in CAD patients, whereas *Coprococcus* and *Klebsiella* were more prevalent in the control group (Fig. 1C). In glycolysis III (from glucose), *Escherichia*, *Klebsiella*, and *Bacteroides* were the predominant contributors (Fig. 1D). The predominant contributors to CDP-diacylglycerol biosynthesis I and II were *Bacteroides*, *Faecalibacterium*, and *Escherichia* (Fig. 1E and F). In the L-isoleucine biosynthesis III pathway, the major contributors were *Prevotella* and *Bacteroides* across all participants (Fig. 1G).

**Fig 2:**
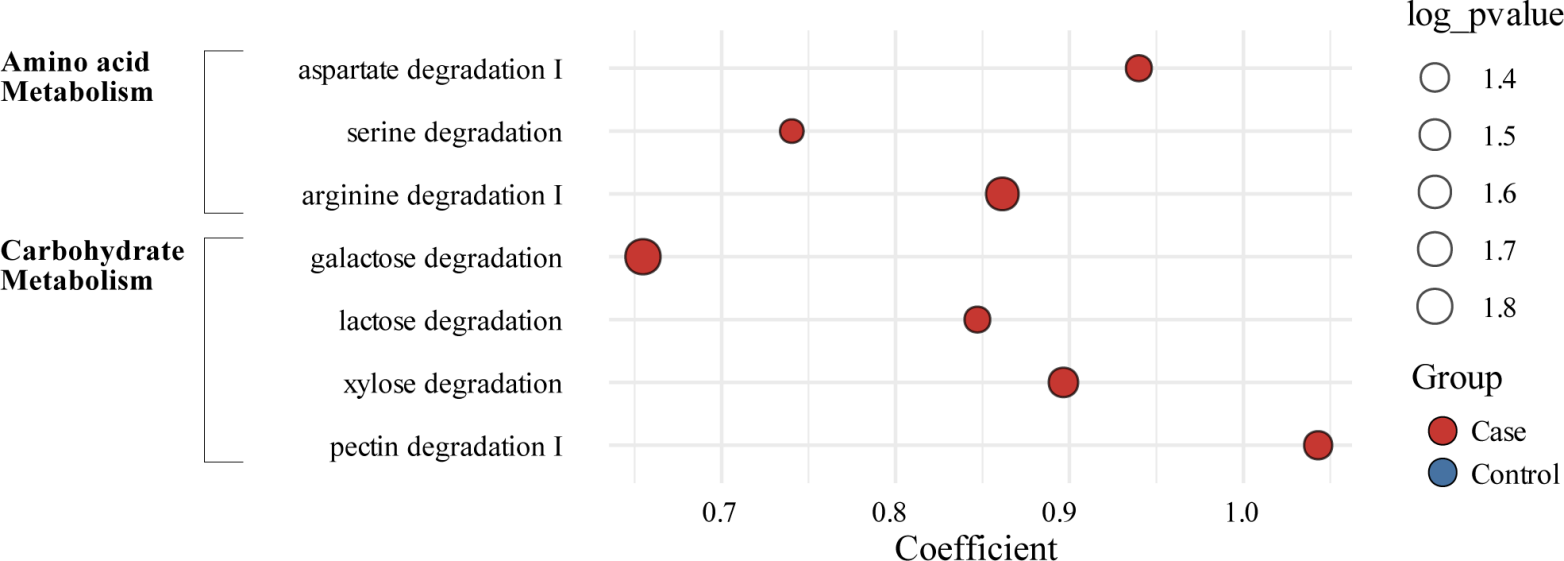
Human gut metabolic modules associated with CAD. Bubble plot for differentially abundant gut metabolic modules (GMMs) between the case and control groups. *P*-values were calculated from differential abundance analysis with MaAsLin2. The size of the bubble is based on *P*-values, where a larger size represents higher significance (lower *P*-value). Red color indicates higher abundance in cases, and blue represents higher abundance in the control group.

To better characterize the functional shifts in the gut microbiome associated with CAD, we analyzed the microbiota metabolic potential and anaerobic fermentation capacity using the human gut metabolic module (GMM) (13). Seven GMMs were significantly enriched in patients with CAD (linear regression model, *P* < 0.05; Fig. 2). Specifically, three amino acid degradation-associated modules (aspartate, serine, and arginine degradation) and four carbohydrate degradation-associated modules (galactose, lactose, xylose, and pectin degradation) were significantly enriched in patients with CAD.

Using UniRef90 profiles from HUMAnN3, we predicted the gut microbial metabolites and identified 80 metabolites. Of these, three metabolites differed significantly between patients with CAD and controls (linear regression model, *P* < 0.05; Table 3). Inosine was significantly elevated in patients with CAD, whereas C18:0e MAG and alpha-muricholate were significantly depleted in patients with CAD compared with those in the controls.

**TABLE 3:** Differentially abundant predicted metabolites in the case and control groups^*a*^

Metabolite	Coefficient	Relative abundance (%)		*P-*value
		Case	Control	
Inosine	0.267	0.009	0.008	2.01E−04
C18:0e MAG	−0.074	0.006	0.006	2.26E−02
Alpha-muricholate	−0.407	0.032	0.042	3.65E−02

^*a*^ *P-*values were generated from the linear regression model using MaAsLin2 between the two groups. Significantly different species were identified. Positive coefficient values indicate higher abundance in the cases compared with controls.

In addition, we examined the relative abundance of bacterial genes encoding enzymes involved in TMA synthesis, including *CutC*, *GrdI,* and *MttB* (Fig. S4). No significant differences were observed between patients with CAD and controls (*P* > 0.05, Wilcoxon rank-sum test).

### Prediction models for CAD

**Fig 3:**
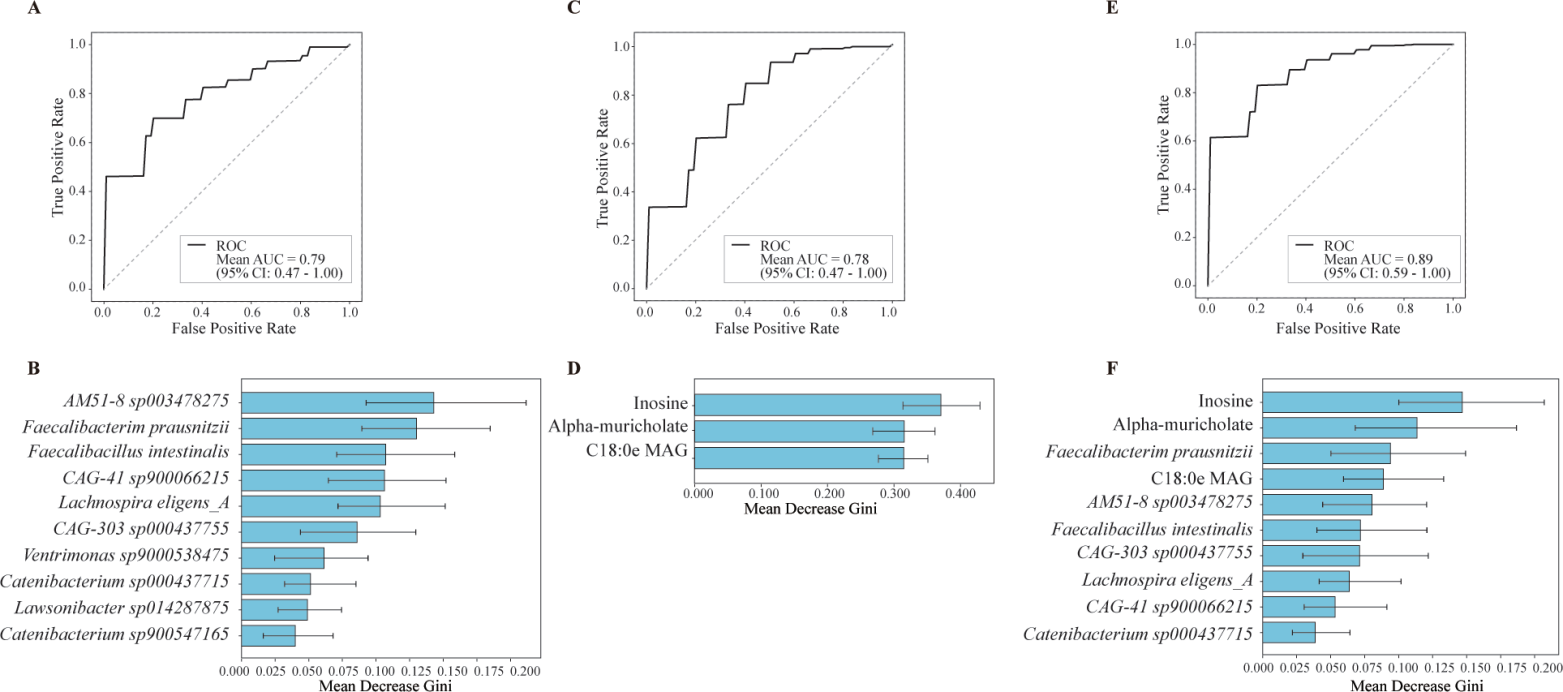
Random forest classification models to distinguish CAD cases from controls. Receiver operating characteristic (ROC) curves based on differentially abundant (**A**) bacterial species, (**C**) predicted microbial metabolites, and (**E**) a combined feature set of both. Feature importance ranked by mean decrease Gini (MDG) for each model, corresponding to (**B**) species-only, (**D**) metabolite-only, and (**F**) combined models. Area under the curve (AUC) and MDG value were evaluated using fivefold cross-validation repeated 50 times. The results are summarized as mean values with 95% confidence intervals across iterations.

To evaluate the predictive potential of the gut microbiota in distinguishing CAD cases from controls, we developed a random forest classification model using differentially abundant bacterial species and metabolites. A classification model based on bacterial species achieved a mean area under the curve (AUC) of 0.79 (95% confidence interval [CI]: 0.47%–1.00%; Fig. 3A), with *AM51-8 sp003478275*, *F. prausnitzii*, and *F. intestinalis* as the top-ranking predictors (Fig. 3B). Similarly, the metabolites-based model yielded a mean AUC of 0.78 (95% CI: 0.47%–1.00%; Fig. 3C), with inosine showing the highest feature importance, followed by alpha-muricholate and C18:0e MAG (Fig. 3D). When bacterial taxa and metabolites were combined, the predictive performance improved, resulting in a mean AUC of 0.89 (95% CI: 0.59%–1.00%; Fig. 3E), highlighting the complementary predictive value of taxonomic and metabolic features. In this combined model, inosine, alpha-muricholate, *F. prausnitzii*, C18:0e MAG, and *AM51-8 sp003478275* were highly important (Fig. 3F). Receiver operating characteristic (ROC) curve analysis indicated that integrating both microbial taxa with metabolites enhanced the predictive accuracy of CAD classification compared with models utilizing a single feature type.

### Identification of MAGs in patients with CAD

MAGs were reconstructed separately for CAD cases and controls using co-assembled contigs (Table S4). A total of 31 bins were reconstructed, with 17 originating from CAD cases and 14 from control participants. In total, 25 distinct MAG species were identified, including six shared between both groups, 11 species found only in CAD cases, and eight species found only in the control group. The six MAGs found in both groups were *Brachyspira aalborgi*, *Megamonas funiformis*, *Sutterella wadsworthensis_A*, *Sutterella wadsworthensis*, *Akkermansia muciniphila,* and *PeH17 sp000435055*. Taxonomic classifications and relative abundances of all MAGs identified in CAD cases and controls are provided in Tables S5 and S6. Among the 25 species identified from co-assembly MAGs, by excluding the six species shared between CAD cases and controls, 18 species overlapped with those detected using MetaPhlAn4, a reference-based taxonomic profiling tool (Fig. S5A).

In addition to the co-assembly approach, we performed individual assemblies for each sample, which resulted in the reconstruction of 326 bacterial MAGs (Table S7). After dereplication at 95% average nucleotide identity (ANI), a total of 144 distinct species-level MAGs were identified through taxonomic classification (Table S8). Among the 25 MAG species identified from co-assembly, 24 MAG species were identified in the individual-assembly-based MAG set, except for *Paraprevotella clara*. Instead, a closely related genome, *Paraprevotella xylaniphila*, which shared 92.62% ANI with *P. clara*, was identified in the individual assembly. Among the 144 MAG species identified through individual assemblies, 96 species overlapped with those detected using MetaPhlAn4 (Fig. S5B). This overlap suggests that a substantial proportion of the MAGs reconstructed in this study correspond to previously characterized microbial species, whereas the remaining MAGs may represent less well-characterized or novel taxa.

**Fig 4:**
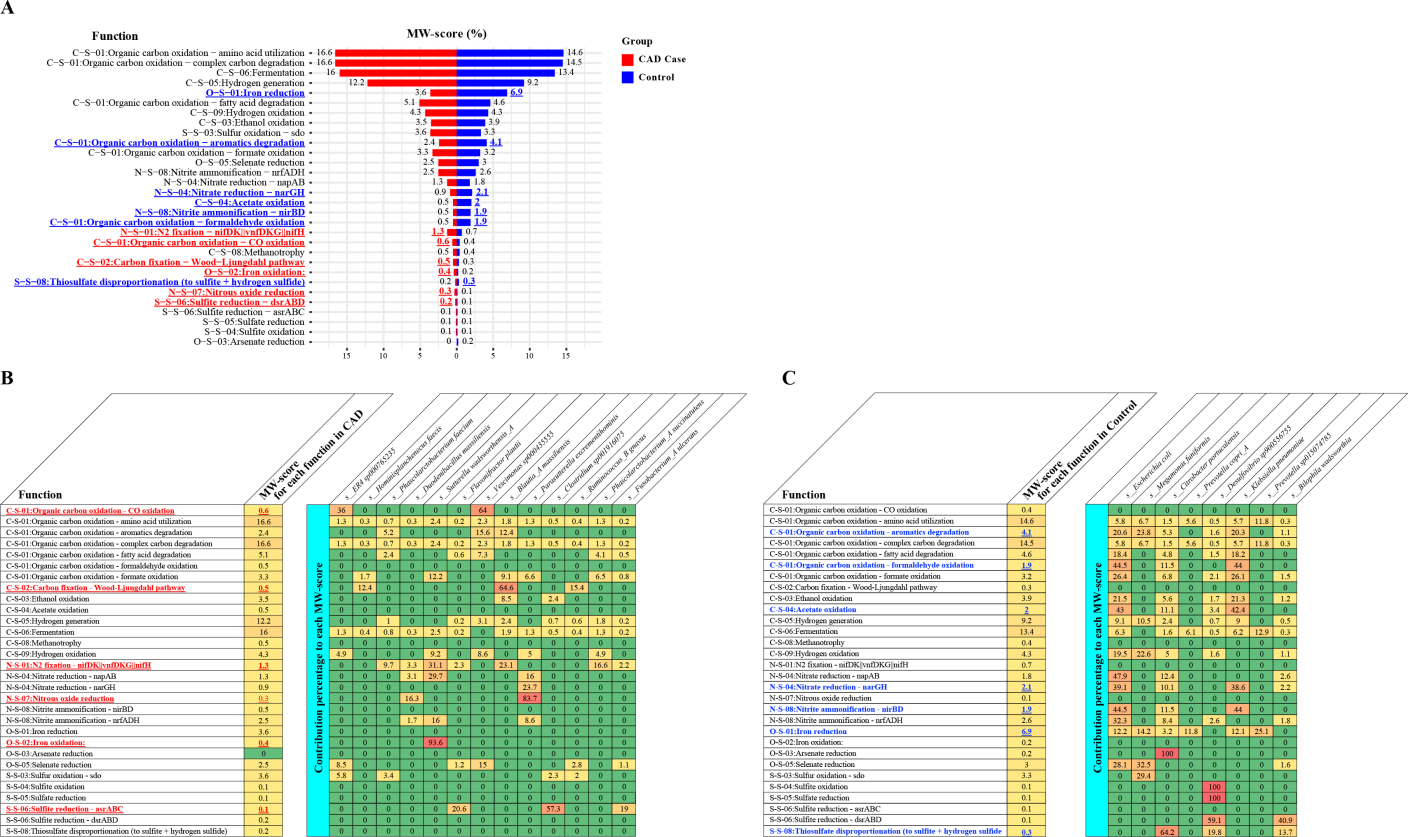
Community-scale metabolic profiling of MAGs using METABOLIC (v4.0). (**A**) Comparison of metabolic weight scores (MW-scores) of individual assembly MAGs between case and control groups. MW-scores represent the proportion of each metabolic function within the total functional capacity of the microbial community. Heatmaps showing species-level contributions to each metabolic function in CAD-derived MAGs (**B**) and control-derived MAGs (**C**), calculated using the METABOLIC-C.pl function.

To assess the potential metabolic interactions within microbial communities, we performed community-scale metabolic profiling of MAGs using metabolic weight scores (MW-scores) (14), which quantify the relative proportion of each metabolic function within the overall functional capacity of the microbial community. In the analysis based on MAGs reconstructed from individual assemblies, MAGs from CAD cases exhibited more than 1.5-fold higher MW-scores for N_2_ fixation, CO oxidation, carbon fixation, iron oxidation, nitrous oxide reduction, and sulfite reduction (Fig. 4A), with *Suterella wadsworthensis, Phascolarctobacterium succinatutens*, and *Clostridium sp001916075* serving as the predominant contributors (Fig. 4B). In contrast, controls-derived individual MAGs showed higher MW scores for aromatic degradation, formaldehyde oxidation, acetate oxidation, nitrate reduction, nitrite ammonification, iron reduction, and thiosulfate disproportionation (Fig. 4C), with major contributors, including *M. funiformis*, *Prevotella copri*, *S. wadsworthensis*, and *Bilophila wadsworthia*. Notably, N_2_ fixation and sulfite reduction were consistently enriched in CAD-derived co-assembly MAGs. Conversely, iron reduction, aromatic degradation, and nitrite ammonification were elevated in control-derived co-assembly MAGs as shown from individual MAGs (Fig. S6).

Furthermore, to assess the genome-scale metabolic potential of MAGs, we examined the functional genes associated with DRAM-annotated metabolic modules (15), lipid metabolism (KEGG: 09103), CVD (KEGG: 09166), and TMA-metabolism (Fig. S7; Table S9). CAD-derived MAGs carried several genes related to ether lipid metabolism (ko00565) or steroid biosynthesis (ko00100/ko00140, Fig. S7A). The TMA-producing gene, *cutC*, was identified from nine MAGs, including six *A. hadrus* MAGs (Case_CM_28, Control_IM_KR_0042_concoct_bin.62, etc.), two *Desulfovibrio sp900556755* MAGs, and one *Bilophila wadsworthia* MAG. Another TMA-producing gene, *cntA/yeaW*, was identified from 10 *M. fumiformis* MAGs (Case_CM_45, Control_CM_39, Control_IM_KR_0032_concoct_bin.15, etc.), one *Klebsiella pneumoniae* MAG, and one *Escherichia coli* MAG. The *mttB (pyl*), non-TMA-producing gene, was identified from three *Phocaeicola* species MAGs from both CAD and control groups. Another non-TMA-producing gene, *mtxB*, was identified from *Phocaeicola plebeius_A* and *F. prausnitzii* MAGs from only the control group.

To evaluate the metabolic features and genetic diversity of the MAGs identified in this study against reference genomes, we conducted a strain-level comparative genomic analysis of four species identified in both case and control MAGs (*B. aalborgi*, *M. fumiformis*, *A. muciniphila*, and *S. wadsworthensis*) using a public database. The control-derived *A. muciniphila* MAGs, control-derived MAGs, Control_CM_12, Control_IM_KR_0033_metabat2_bin.50, and Control_IM_KR_0039_concoct_bin.28, carried functional modules for arabinan and xyloglycan degradation, compared with the CAD-derived *A. muciniphila* MAGs, Case_CM_16 and Case_IM_KR0014_metabat2_bin.41 (Fig. S8). The CAD-derived *M. fumiformis* MAG, Case_CM_45, displayed carriage of metabolic modules related to methanogenesis, mercury reduction, acetate conversion, and lipid metabolism compared with those from eight control-derived MAGs, including Control_CM_39, Control_IM_KR_0033_concoct_bin.53, and Control_IM_KR_0031_metabat2_bin.41 (Fig. S9). No significant differences were found between the CAD and control MAGs in terms of *B. aalborgi* and *S. wadsworthensis* (Fig. S10 and S11).

## DISCUSSION

This study provides a comprehensive analysis of gut microbiota composition, metabolic pathways, predicted microbial metabolites, and MAGs in individuals with CAD using shotgun metagenomic sequencing. These findings contribute to our understanding of gut microbial alterations in patients with CAD and offer potential implications for microbiome-based therapeutic interventions. Notably, high AUC values in predictive models indicate that gut microbial markers may be clinically relevant for noninvasive CAD risk stratification. However, further validation in larger cohorts and longitudinal studies is required to confirm these findings and assess their applicability in clinical settings.

We identified 15 bacterial species with differential abundance between patients with CAD and controls, underscoring the potential taxonomic shifts associated with CAD. Several members of the Lachnospiraceae family were significantly enriched in the CAD cases, whereas *Lachnospira eligens A* was depleted. Previous studies have reported a reduction in Lachnospiraceae in CAD, which is often associated with lower SCFA production (16) and increased TMAO levels (3), both of which contribute to the progression of atherosclerosis. However, our findings suggest an enrichment of distinct Lachnospiraceae taxa, indicating a more nuanced role for this family in CAD. In contrast, well-characterized SCFA-producing bacteria, such as *F. prausnitzii*, *Catenibacterium* spp., and *Holdemanella* spp., were consistently depleted in CAD. *F. prausnitzii*, a well-known butyrate producer, plays a key role in maintaining intestinal barrier integrity and modulating colonic inflammation (17). Its reduction in patients with CAD was consistent with previous findings and underscores its protective role in cardiovascular health (18). Furthermore, the depletion of *Catenibacterium* spp. and *Holdemanella* spp., which are both linked to anti-inflammatory and metabolic benefits (19), suggests that the loss of SCFA-producing bacteria may contribute to metabolic dysregulation and systemic inflammation in CAD. These findings highlight the gut–heart axis and therapeutic potential of restoring SCFA-producing bacteria in CAD management.

Additionally, *S. isoflavoniconvertens*, a bacterium that converts dietary isoflavones into equol, is significantly reduced in patients with CAD. Equol has been linked to anti-inflammatory and cardioprotective properties, and its depletion may contribute to impaired cardiometabolic homeostasis (20). Similar reductions in *S. isoflavoniconvertens* have been reported in lean non-alcoholic fatty liver disease, further supporting its potential role in metabolic disorders (21). *P. copri* (recently renamed *Segatella copri*), a member of the Prevotellaceae family (formerly classified in Bacteroidaceae family), was significantly reduced in patients with CAD. These findings were consistent with other studies reporting the depletion of *Prevotella* and *Bacteroides* species in patients with atherosclerotic CVD (22). The depleted *P. copri* has also been reported to be associated with favorable cardiometabolic profiles, such as lower visceral fat, higher polyunsaturated fatty acids, and reduced C-peptide (23). Notably, *P. vulgatus*, a member of *Bacteroidaceae*, has been associated with protection against atherosclerosis by reducing microbial LPS levels and maintaining barrier function (24), although no significant reduction of *Phocaeicola* species in CAD patients was identified in this study. *Bacteroidaceae* contribute to gut barrier integrity by stimulating mucus production and supporting intestinal epithelial function (25). Disruption of this barrier may allow microbial components such as peptidoglycan and flagellin to translocate into the circulation, promoting systemic inflammation and atherosclerosis (26). These results imply that microbiome-regulated gut barrier health may be a key pathophysiological mechanism in CVD.

Gut microbial metabolic pathways provide critical insights into the pathogenesis of CAD. In this study, we observed a significant enrichment of the urea cycle and L-citrulline biosynthesis in patients with CAD. The urea cycle, which is involved in ammonia detoxification, is linked to CVD severity and mortality via mechanisms related to inflammation and myocardial energetics (27–29). The L-citrulline biosynthesis pathway, primarily driven by *Alistipes* spp., was enriched in CAD cases. Although L-citrulline supplementation has shown blood pressure-lowering effects, recent evidence suggests that it may increase blood pressure during exercise, indicating context-dependent effects (30, 31). Conversely, the depletion of L-isoleucine biosynthesis and BCAA super pathways, which are crucial for muscle nitrogen balance and energy metabolism (32), in CAD cases is consistent with previous findings (33), further supporting its potential contribution to metabolic stress and CAD progression.

The glycolysis III pathway, primarily driven by Enterobacteriaceae family members, such as *E. coli, K. pneumoniae,* and *B. fragilis*, was enriched in CAD cases. As opportunistic pathogenic bacteria, Enterobacteriaceae may utilize glycolysis to produce inflammatory mediators, such as enterotoxins, promoting systemic inflammation, vascular dysfunction, and the progression of CVD (22). In addition, CDP-diacylglycerol synthesis, a precursor for key phospholipids, such as phosphatidylglycerol and cardiolipin (34), was also enriched in CAD. Bacterial cardiolipin has been linked to inflammation and cell death via Toll-like receptor signaling during host metabolism (35). Interestingly, while these pathways were elevated in our CAD cohort, a previous study reported their depletion in CAD patients (36). These contrasting results suggest that additional factors, such as microbial gene expression, host metabolism, and environmental factors, may play crucial roles in microbial functional shifts in CVD, underscoring the need for cross-cohort validation.

Regarding human gut metabolic functions, CAD cases showed a greater potential for degrading amino acids, particularly arginine and aspartate. This aligns with previous findings showing the enrichment of arginine and serine degradation pathways in patients with CVD, largely driven by pathogenic microbiota (37). The increased microbial conversion of arginine to ornithine and urea may impair NO bioavailability, potentially impairing vascular function and increasing CVD risk (38); in addition to amino acid degradation, patients also have a higher capacity for simple carbohydrate degradation, particularly lactose and xylose. Enrichment of xylose, galactose, and lactose degradation pathways has been associated with an increased production of pro-inflammatory metabolites in patients with CVD and diabetic CAD (39, 40). Collectively, excessive amino acid degradation and the fermentation of simple carbohydrates may exacerbate intestinal barrier dysfunction and contribute to cardiovascular deterioration.

We identified three differentially abundant predicted gut metabolites in patients with CAD. The inosine levels were significantly elevated, which is consistent with previous studies linking inosine to CAD and acute myocardial infarction (41). As a coronary vasodilator, inosine plays a role in relaxing the coronary arteries; however, its precise mechanism remains unclear. Inosine has been implicated in the development of targeted therapeutics for CVDs, cancer, inflammation, and neurological conditions (42). However, its potential role in CVD therapeutics and inflammatory modulation warrants further investigation. C18:0e MAG, a monoacylglycerol phospholipid, is depleted in patients with CAD; however, its role in cardiovascular health remains unknown. Although alpha-muricholic acid was also depleted, its function in the human gut microbiome and its association with CAD require further study.

Random forest classification model analysis highlights the predictive relevance of the gut microbiota and their metabolic products in CAD. Notably, combining differentially abundant bacterial species with predicted microbial metabolites improved the classification performance (area under the curve [AUC] = 0.89) compared with that of models based on either feature alone. This suggests that taxonomic and metabolic information provide complementary insights into the host–microbiome interactions underlying CAD. Among the top predictors, *AM51-8 sp003478275* and *F. prausnitzii* have been previously implicated in metabolic dysregulation and systemic inflammation in CAD (17), whereas metabolites, such as inosine, have been associated with coronary vasodilation (41). These findings support the growing evidence that integrated microbiome features can serve as robust biomarkers for CAD and underscore the potential of multi-omics approaches for microbiome-based diagnostics in CVD.

Using metagenomic assembly and binning, CAD-derived MAGs showed elevated MW-scores for N_2_ fixation and sulfite reduction pathways. Enrichment of N_2_ fixation could indicate a shift toward microbial self-sufficiency in nitrogen acquisition, potentially compensating for disrupted nitrogen metabolism in the host gut environment (43). Likewise, increased sulfite reduction might be linked to altered sulfur metabolism and increased production of hydrogen sulfide, a gasotransmitter implicated in cardiovascular dysfunction and mucosal inflammation (44). Conversely, control-derived MAGs were enriched in iron reduction, aromatic compound degradation, and nitrite ammonification. Enhanced aromatic degradation may suggest a gut microbiota more equipped for processing complex dietary polyphenols and aromatic compounds, which have been associated with cardioprotective effects (45). Furthermore, increased iron reduction and nitrite ammonification may reflect a redox-balanced and metabolically versatile microbial community that favors stability over inflammatory triggers (46, 47). These findings suggest that metabolic restructuring of the gut microbiome may play a key role in the ecological adaptation of microbes to host cardiovascular health status.

Interestingly, our strain-level comparative genomic analysis revealed distinct functional profiles between the CAD-derived and control-derived MAGs. Both *M. fumiformis* and *A. muciniphila* have been reported as potentially protective taxa in CVD pathophysiology (48, 49). However, the CAD-derived *M. fumiformis* strain showed carriage of functional modules for acetate-centered metabolism (methanogenesis/methanotroph, SCFA conversion), mercury reduction, and fatty acid degradation, indicating a shift toward pro-atherogenic metabolism (50, 51), compared with those in control-derived MAGs. Additionally, *A. muciniphila* MAGs from controls carried more functional modules for complex carbohydrate degradation (e.g., arabinan, xyloglucan), potentially mucosal protective and anti-inflammatory properties (48). However, given that these interferences from gene presence/absence, not capturing expression, regulation, enzyme activity, or physiological context, further experimental validation seems to be needed to prove their biological relevance. These findings underscore the critical importance of strain-level functional heterogeneity, as revealed through MAG-based analysis, in shaping host–microbe interactions and influencing CAD pathophysiology.

Despite the well-established link between TMAO and CVD (52), our study found no significant differences in TMAO-related gene abundances between CAD and control groups when mapping individual fastq samples to the TMA-related gene database. Consistently, Griffin et al. highlighted that choline oxidation and TMAO production were not predictive of atherosclerotic plaque formation in animal models. (53, 54). However, genome-resolved MAGs analysis in the present study has provided deeper insights into the strain-specific distribution of TMA-related genes. Notably, the *cutC* gene (55), responsible for choline-to-TMA conversion, was detected in *A. hadrus* MAGs from both CAD and control groups, whereas the *cntA/yeaW* gene (56, 57), associated with TMA production from ammonium compounds like carnitine and γ-butyrobetaine, was present in *M. fumiformis* MAGs from both CAD and control groups. Although both *A. hadrus* and *M. fumiformis* are generally regarded as SCFA producers (49, 58), our MAG-based analysis revealed carriage of TMA-producing genes in their genomes, suggesting a potential dual role in gut-host interactions. On the other hand, among the reported non-TMA-producing genes, *mtxB* (quaternary amine methyltransferase) (59) was detected in *P. plebeius_A* and *F. prausnitzii* species MAGs from only the control group, while *mttB* (trimethylamine methyltransferase) (60) was detected in *Phocaeicola* species MAGs from both CAD and control groups. *Phocaeicola* species and *F. prausnitzii* are well recognized for their anti-inflammatory traits (17). Given that *F. prausnitzii* was significantly depleted in CAD cases in our MetaPhlAn4-based differential abundance analysis, this observation suggests that *F. prausnitzii* could play a potential protective role in CAD pathophysiology. Together, these findings highlight the value of MAG-based approaches for uncovering strain-level functional heterogeneity that may be obscured in community-level analyses. Therefore, it is important to elucidate the microbial contributions to TMAO metabolism and their subtle roles in CAD pathogenesis.

Despite these novel findings, this study had some limitations. First, the relatively small and sex-imbalanced sample size may limit the robustness and generalizability of the findings despite the use of PSM to reduce confounding factors. Second, the cross-sectional design precludes causal inference, highlighting the need for longitudinal studies to clarify microbial dynamics in CAD progression. Third, while shotgun metagenomics enables strain-level resolution and provides valuable insights into the metabolic potential of microbial communities, it has limited capacity to capture the *in situ* activity of these pathways. Future studies integrating metatranscriptomics, metaproteomics, or metabolomics will be needed to directly assess functional activity and host-microbe interactions. Despite these limitations, this is the first study to report the discovery of distinctive bacterial MAGs in patients with CAD and healthy controls, offering novel insights into the role of the gut microbiome in the pathophysiology of CAD.

In conclusion, this study provides a comprehensive characterization of the gut microbial signature associated with CAD using shotgun metagenomics. These findings underscore the importance of gut microbial and functional profiling in understanding CAD and provide a foundation for microbiome-targeted therapeutic strategies for CAD management and risk reduction. Future studies should focus on validating these microbial signatures in larger cohorts and exploring their mechanistic roles in cardiovascular health.

## MATERIALS AND METHODS

### Study subjects, data collection, and group definition

This cross-sectional study was part of the Kangbuk Samsung Cohort Study involving 1,710 Korean adults (aged 23–77 years) who underwent regular health screening between 2014 and 2021 (61). Medical histories, medication use, and lifestyle factors were assessed using structured questionnaires. Anthropometrics, blood pressure, and blood samples were analyzed using standardized protocols. The methodological details of data collection are provided in the Supplemental Methods. After excluding individuals based on recent antibiotic use, antacid use, history of cancer, or history of stroke, 1,585 participants remained (Fig. S1A).

CAD was diagnosed in individuals with a documented history of angina pectoris, myocardial ischemia, or angiographically confirmed stenosis (>50%) in at least one major coronary artery (33). To minimize the imbalance between patients with CAD and controls, PSM was performed using age, sex, and BMI as covariates. In total, 42 participants (14 CAD cases and 28 controls) were included in the final matched analysis (Fig. S1B). The methodological details of PSM are provided in the Supplemental Methods.

### DNA extraction and shotgun metagenomic sequencing

Detailed methods for fecal sample collection, metagenomic DNA extraction, shotgun metagenomic sequencing, and raw metagenomic data pre-processing are provided in the Supplemental Methods.

### Taxonomic and functional profiling of metagenomic data

For taxonomic classification, metagenomic reads were analyzed using MetaPhlAn4 (v.4.0.6) (62) with default parameters. Only the microbial species present in at least 10% of the samples were included in the analysis to mitigate the impact of rare taxa (63). Functional profiling was performed using HUMAnN3 (v.3.0) (64). To visualize species-level contributions to metabolic pathways, stratified relative abundance bar plots were generated using the humann_bar plot function in HUMAnN3 (64). MelonnPan was used to predict microbial metabolites with the UniRef90 gene profile as the input data (65). Functional genes associated with human gut metabolites were analyzed using the curated GMM database (13) using Omixer-RPM (v0.3.2) (66), with a 33.3% minimum coverage threshold. Taxonomic profiles, functional genes, and GMM modules were all calculated as relative abundances. TMA-related genes were quantified based on reads per kilobase per million mapped reads (RPKM) with the Methylated Amine Gene Inventory of Catabolism database (MAGICdb) (67) using CoverM (v.0.7.0) (68).

### MAG reconstruction and phylogenetic analysis

To reconstruct MAGs, we performed both co-assemblies for the CAD and control groups and individual assemblies for all samples. All high-quality non-human reads were pooled for each group and assembled independently using MEGAHIT (v.1.2.9) (69), excluding contigs shorter than 1 kb. Binning of the assembled contigs was performed using three independent binning algorithms: MaxBin2 (70), MetaBAT2 (71), and CONCOCT (72). The resulting bins were refined using DAStool (73) to obtain near-complete genomes with minimal contamination. Genome quality was assessed using CheckM (74), applying strict thresholds of >90% completeness and <10% contamination for the final bin selection (Tables S4 and S7). The taxonomic classification of MAGs was determined using GTDB-tk (v2.0) (75) from the GTDB database (v.214). Dereplication of individual MAGs was performed at thresholds of 95% ANI with dRep (v.3.4.2) (76). Relative abundances of both co-assembly MAGs and dereplicated individual MAGs across samples were quantified using CoverM (v.0.7.0).

To assess potential metabolic interactions within microbial communities, we utilized METABOLIC (v4.0) (14) for community-scale metabolic profiling of MAGs using MW-scores, representing the relative contribution of each function to the total community capacity. The species-level contributions to each metabolic pathway were calculated using the METABOLIC-C.pl function in METABOLIC (14). Furthermore, to investigate the genome-wide functional potential, we annotated MAGs based on DRAM-implemented functional modules (15), lipids (KEGG: 09103), CVD (KEGG:09166)-associated KEGG orthologs (77), and MAGICdb-implemented TMA-related genes (67). Phylogenetic trees were constructed using PhyloPhlAn (v.3.0) (78). The iTol web server (v.7, https://itol.embl.de/) (79) was used to visualize the functional analysis of MAGs.

To evaluate the metabolic features and genetic diversity of MAGs identified in this study against reference genomes, we conducted strain-level comparative genomic analysis for four species (*B. aalborgi*, *M. fumiformis*, *A. muciniphila*, and *S. wadsworthensis*), identified in both case and control MAGs as well as individual MAGs, using a public database, the Bacterial and Viral Bioinformatics Resource Center (BV-BRC) (80). Whole genome sequences of human-derived bacterial species with available metadata (e.g., country of origin and year of isolation) were utilized, totaling 34 *B. aalborgi*, 53 *M*. *fumiformis*, 204 *S*. *wadsworthensis*, and 429 *A*. *muciniphila* strains (accessed on 25 May 25 2025; Table S10). Comparative genomic analyses were conducted using the same pipeline as that used for MAGs identified in this study.

### Statistical analysis

Alpha and beta diversity indices were computed to assess microbial community structure using the vegan R package (v.2.6.4) (81). Alpha diversity (Chao, Simpson, Shannon, and Pielou’s evenness) was calculated, and group differences were assessed using the Wilcoxon rank-sum test. Beta diversity was computed based on Bray–Curtis dissimilarity, and intergroup significance was tested using PERMANOVA with 999 permutations.

To identify group differences in microbial taxa, MetaCyc metabolic pathways, GMMs, and predicted metabolites, we applied generalized linear models using the Microbiome Multivariable Association with Linear Models 2 (MaAsLin2) (82). In the MaAsLin2 models, we estimated the coefficients of log-transformed relative abundance by comparing the CAD group with the reference control group. Differential abundance of TMA-related genes was assessed using the Wilcoxon rank-sum test.

To assess the predictive potential of microbial features in distinguishing patients with CAD from controls, we applied a random forest classification model using three feature sets: the differential abundances of (i) bacterial species (ii), predicted microbial metabolites, and (iii) both bacterial species and metabolites. The model performance was evaluated using fivefold cross-validation (500 decision trees per model, 50 iterations). The ROC curves and AUC values were computed across iterations. The feature importance was determined using the mean decrease Gini index. The results are summarized as mean values across iterations with 95% CIs. Predictive model analyses were performed using Python (v3.10.18) with Pandas (v2.3.0), sklearn (v1.7.0), and matplotlib (v3.10.3).

## Data Availability

Raw sequencing data generated in this study have been deposited in the NCBI Sequence Read Archive under accession number PRJNA1195215. Analysis codes, intermediate files, and STORM checklists are publicly available at Zenodo (https://doi.org/10.5281/zenodo.17250068) and are also provided in the Supplemental Material.

## SUPPLEMENTAL MATERIAL

Supplemental Material msystems.00954-25-s0001.pdf Supplemental methods; Fig. S1 to S11.

Supplemental Tables msystems.00954-25-s0002.xlsx Tables S1 to S10.

ASM does not own the copyrights to Supplemental Material that may be linked to, or accessed through, an article. The authors have granted ASM a non-exclusive, world-wide license to publish the Supplemental Material files. Please contact the corresponding author directly for reuse.
