# Supplementary material for: Metagenome-assembled genomes reveal microbial signatures and metabolic pathways linked to coronary artery disease
Source: mSystems. 2025 Nov 6;10(12):e00954-25. doi: 10.1128/msystems.00954-25 (PMC12710354; doi:10.1128/msystems.00954-25)
Supplement: Supplemental Material — Supplemental methods; Fig. S1 to S11. [file msystems.00954-25-s0001.pdf]

Metagenome-assembled genomes reveal microbial signatures and metabolic pathways linked to coronary artery disease

Running title: Gut microbial signature in coronary artery disease

Soomin Lee[a], Shahbaz Raza[a], Eun-Ju Lee[a], Yoosoo Chang[a,b,c], Seungho Ryu[a,b,c], Hyung-Lae Kim[d], Si-Hyuck Kang[e,f], Han-Na Kim[a,g,*]

[a] Department of Clinical Research Design and Evaluation, Samsung Advanced Institute for Health Sciences and Technology, Sungkyunkwan University, Seoul, Republic of Korea

[b] Center for Cohort Studies, Total Healthcare Center, Kangbuk Samsung Hospital, Sungkyunkwan University School of Medicine, Seoul, Republic of Korea

[c] Department of Occupational and Environmental Medicine, Kangbuk Samsung Hospital, Sungkyunkwan University School of Medicine, Seoul, Republic of Korea

[d] Department of Biochemistry, College of Medicine, Ewha Womans University, Seoul, Republic of Korea

[e] Cardiovascular Center, Seoul National University Bundang Hospital, Seongnam-si, Korea

[f] Department of Internal Medicine, Seoul National University, Seoul, Korea

[g] Center for Clinical Epidemiology, Samsung Medical Center, Sungkyunkwan University, Seoul, Republic of Korea

*** Correspondence:**

Han-Na Kim

Address: 115, Irwon-ro, Gangnam-gu, Seoul, Republic of Korea

E-mail: hanna147942@gmail.com; Tel: +82-2-3410-2427, Fax: + 82-2-3410-6639

Supplementary Methods

Study subjects, data collection and group definition

Data regarding medical history, medication use, smoking status, alcohol use, and sociodemographic characteristics were obtained through a self-administered, structured questionnaire. Dietary intake was assessed using a 103-item food frequency questionnaire (FFQ) developed and validated for the Korean population (1), which was designed to measure daily food consumption in the previous year. During health examinations, trained hospital staff performed standardized measurements of blood pressure and anthropometric parameters. Body mass index (BMI) was calculated as body weight (kg) divided by the square of height (m^2). Blood samples were collected after at least 10 hours of fasting. Serum levels of triglyceride (TG), low high-density lipoprotein cholesterol (HDL-C), glucose, high-sensitivity C-reactive protein (HCRP), and C-reactive protein (CRP) were quantified using a homogeneous enzymatic colorimetric assay. Systolic and diastolic blood pressure (SBP and DBP, respectively) were measured using automated sphygmomanometers.

Given the substantial imbalance in the number of cases and controls, propensity score matching (PSM) was performed to select an appropriate control group and achieve a balanced comparison. Propensity score values were estimated using a logistic regression model, considering age, sex, and body mass index (BMI) as covariates. Four matching methods—nearest neighbor, optimal, full, and genetic matching—were evaluated. The genetic matching method was selected as it minimized the average standardized mean difference (SMD) of confounding factors before and after matching (Figure S1B). For PS matching, we employed R packages MatchIt (v.4.5.5),

lmtest (v.0.9-40), smd (v.0.6.6), and cobalt (v.4.5.3). Following PSM in a 1:2 ratio, 42 participants (14 cases and 28 controls) were included in the final matched samples for further analysis.

DNA extraction and shotgun metagenomic sequencing

Fecal samples were collected from participants, immediately frozen at −20°C upon defecation, and subsequently stored at −70°C within 24 hours until further processing. The OMNIgene-GUT collection kit (OMR-200, DNA Genotek, Ottawa, Canada) was used for sample collection. Microbial genomic DNA was extracted within one month of storage using the DNeasy PowerSoil Pro Kits (Qiagen, Hilden, Germany), following the manufacturer's protocol. The concentration and quality of the extracted DNA were measured using a Qubit fluorometer (Invitrogen, CA, USA), and the DNA was stored at −70°C until further experiment. To minimize sequencing batch effects, DNA samples were stored after extraction until all samples were collected, and then library preparation and sequencing were performed in a single batch. High-throughput shotgun metagenomic sequencing was conducted on the Illumina NovaSeq 6000 platform (2 x 150 bp paired-end reads). Raw sequencing reads were preprocessed by removing adapter sequences and low-quality reads using Trimmomatic (v.0.39) (2). Host-derived sequences were filtered by mapping all metagenomic reads to the human reference genome (hg38) using BWA (v.0.7.17) (3) , and non-human DNA reads were extracted using Samtools (v.1.15.1) (4).

Supplementary Tables

Supplementary tables are presented in the excel file named "Supplementary_tables_R2_fn.xlsx".

Index

Table S1. Basic characteristics of all the participants in the study

Table S2. Metadata of metagenomic samples

Table S3. Relative abundance table of all taxonomic species identified from MetaPhlAn4

Table S4. Quality assessment results of the co-assembly MAGs

Table S5. Relative abundance (%) of co-assembly MAGs found in the cases.

Table S6. Relative abundance (%) of co-assembly MAGs found in the control samples.

Table S7. Quality assessment results of the individual-assembly MAGs

Table S8. Relative abundance (%) of individual-assembly MAGs across all samples

Table S9. Genome-wide comparative genomic analysis of individual-assembly MAGs

Table S10. Metadata of public database strains

Supplementary Figures

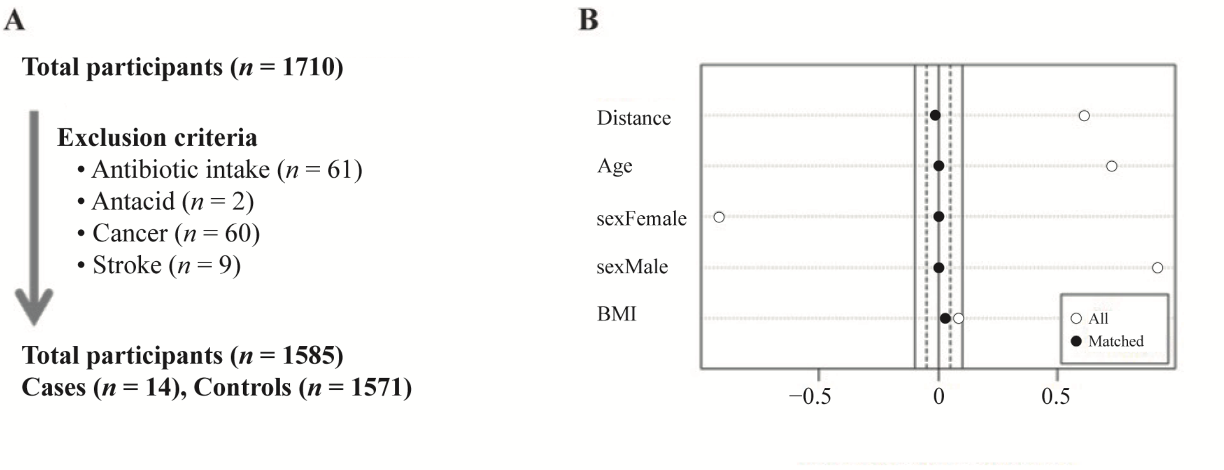

Fig. S1. Sample selection for the study. **(A)** Exclusion criteria used in the study. **(B)** Standardized mean difference between the matched and unmatched samples. The mean difference was calculated using a genetic-based propensity scoring model. Following PS matching in a 1:2 ratio, 42 participants (14 cases and 28 controls) were included in the final matched samples for further analysis.

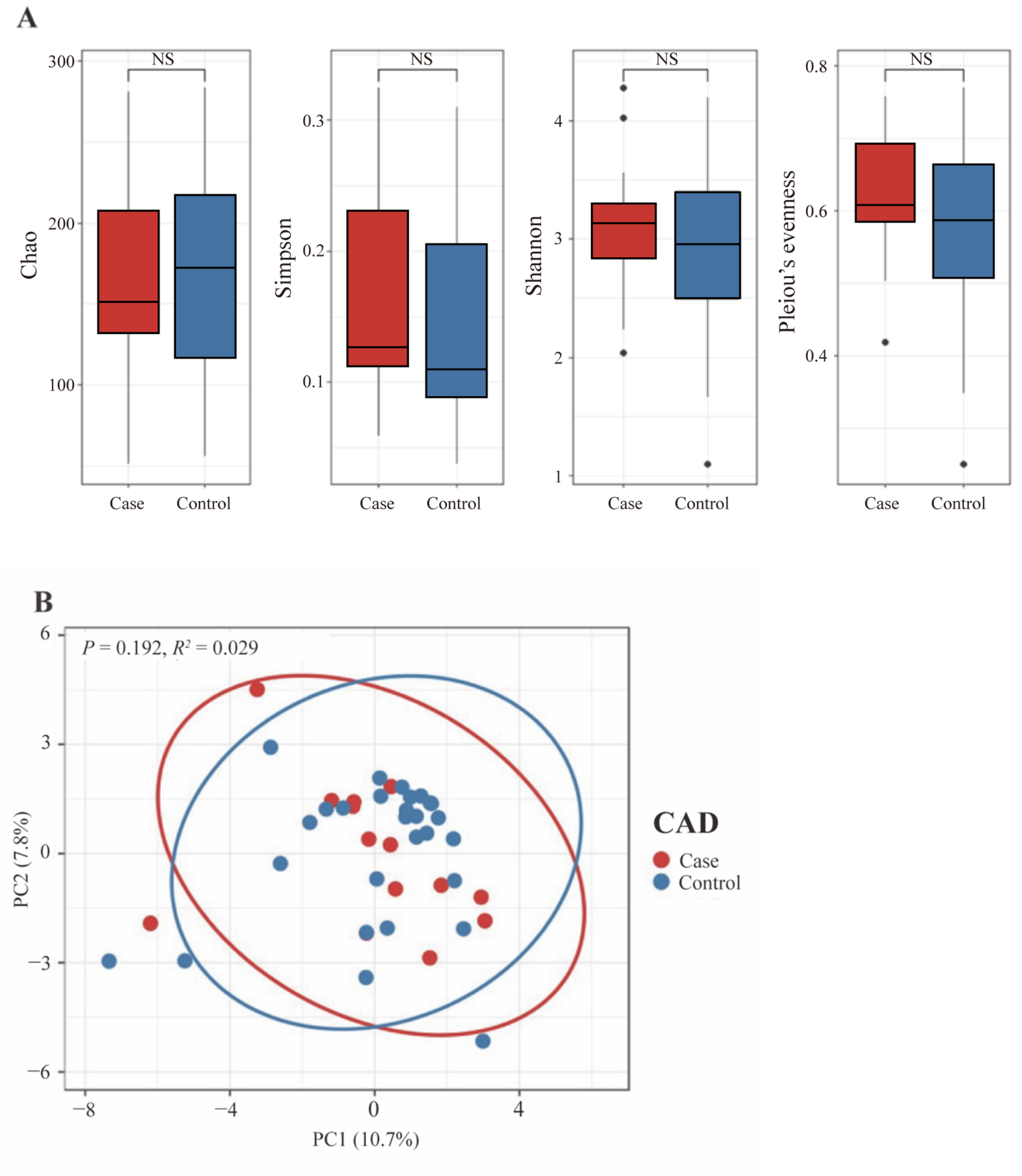

Fig. S2. Alpha diversity analysis for coronary artery disease (CAD) case and control groups was based on the relative abundance of bacterial taxa **(A)**. Principal component analysis (PCA) of the microbiome structure in all samples **(B)**. The Wilcoxon rank-sum test was used for the comparative analysis of alpha diversity between the groups. PCA was based on the Bray–Curtis distance matrix and *p-values* were calculated using a pairwise permutational multivariate analysis of variance (PERMANOVA) with 999 random permutations. Abbreviations. CAD, coronary artery disease; NS, non-significant.

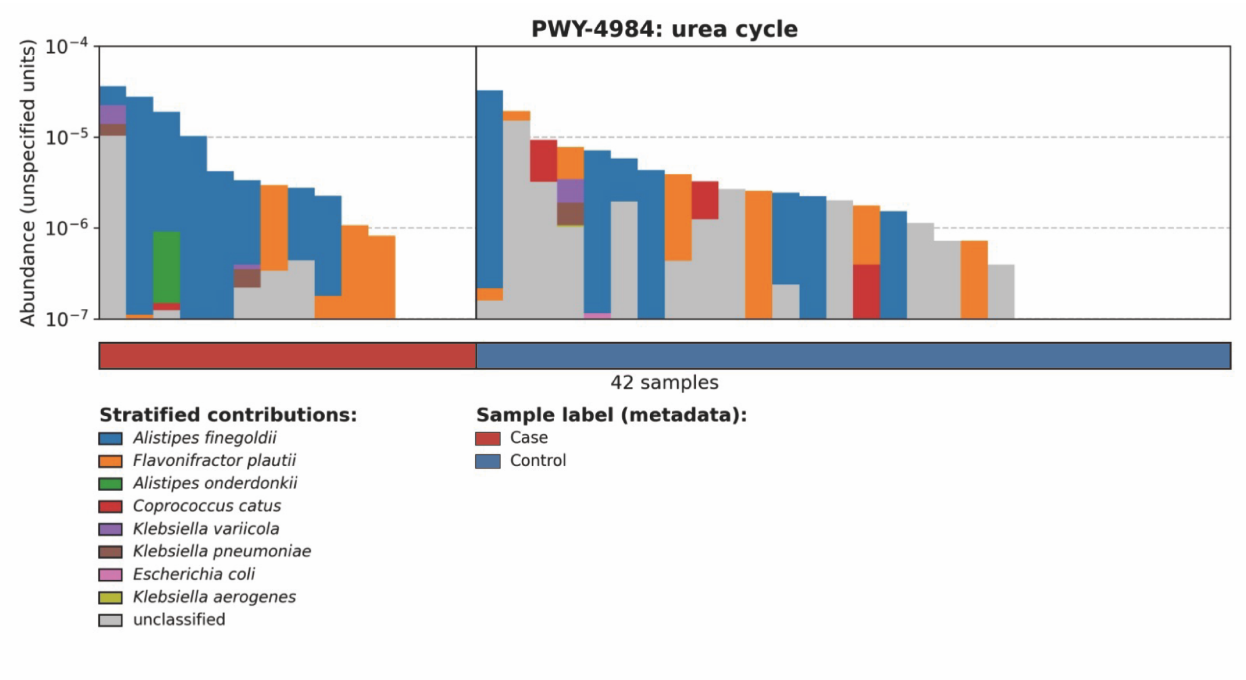

Figure S3. The bar graph shows the contributions of the bacterial species to the urea cycle.

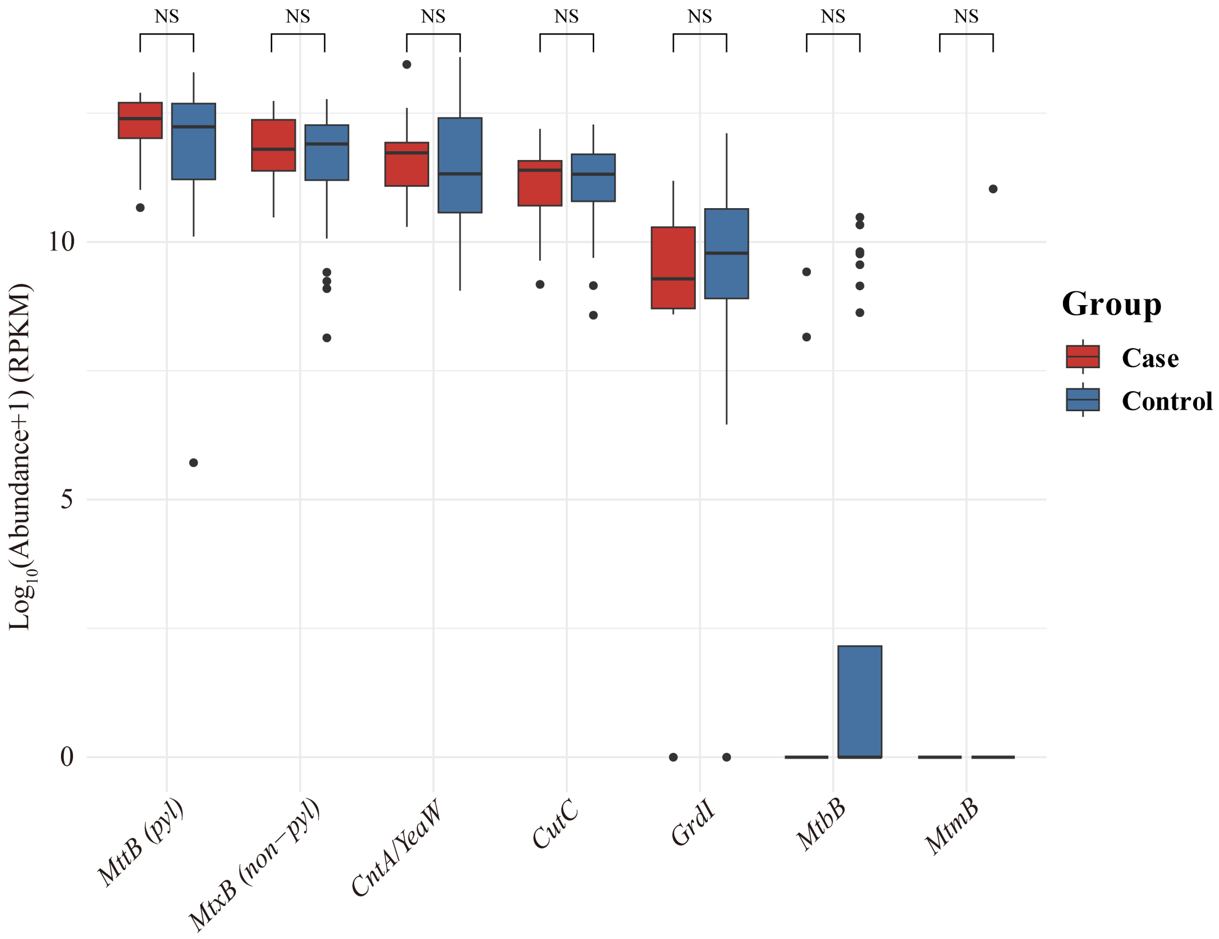

Fig. S4. Bar graph for trimethylamine (TMA)-related genes in CAD cases and controls. The Wilcoxon rank-sum test was used for the comparative analysis of the abundance of TMA-related genes between groups. *$p < 0.05$. CAD, coronary artery disease; RPKM, Reads Per Kilobase per Million mapped reads.

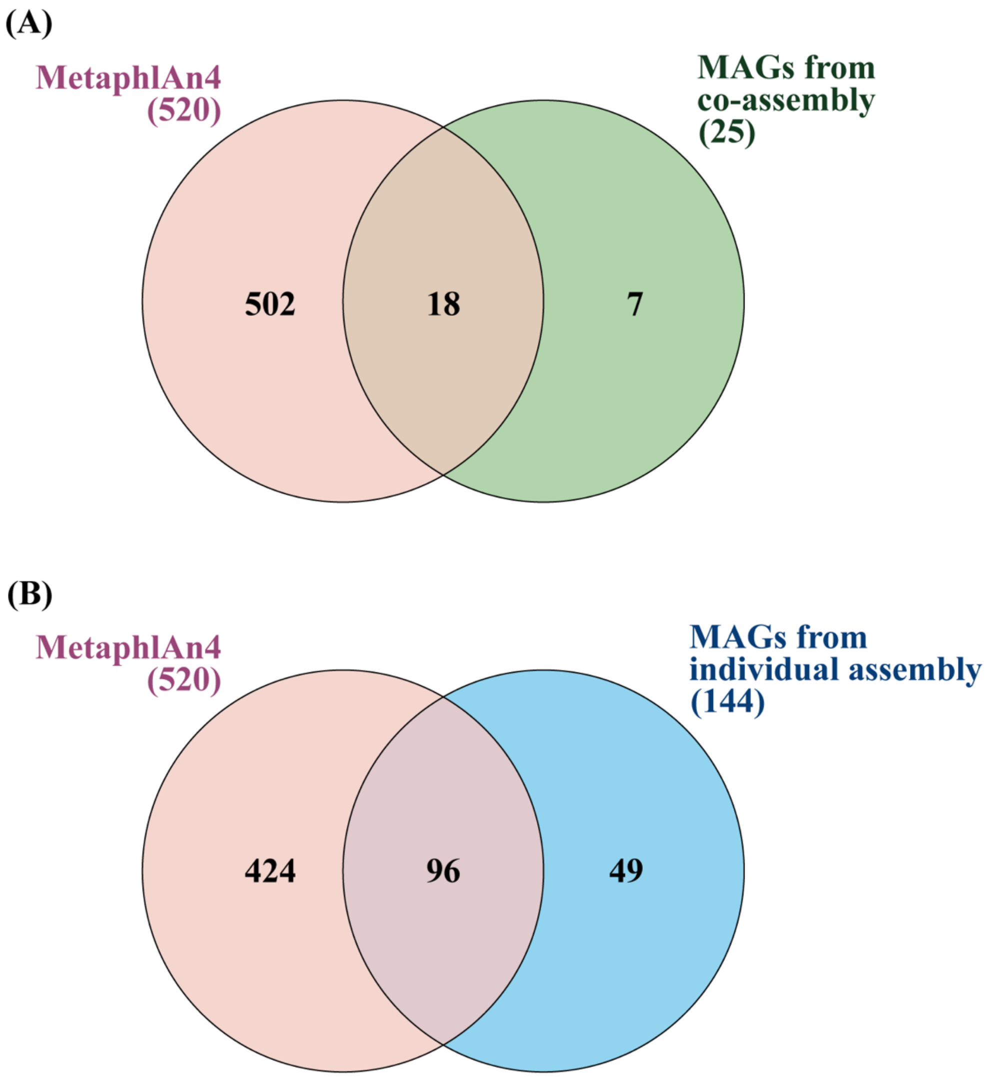

Fig. S5. Venn diagram of shared species observed between MetaPhlAn4 and metagenome-assembled genome (MAG) analysis from co-assembly (A) and individual assembly (B).

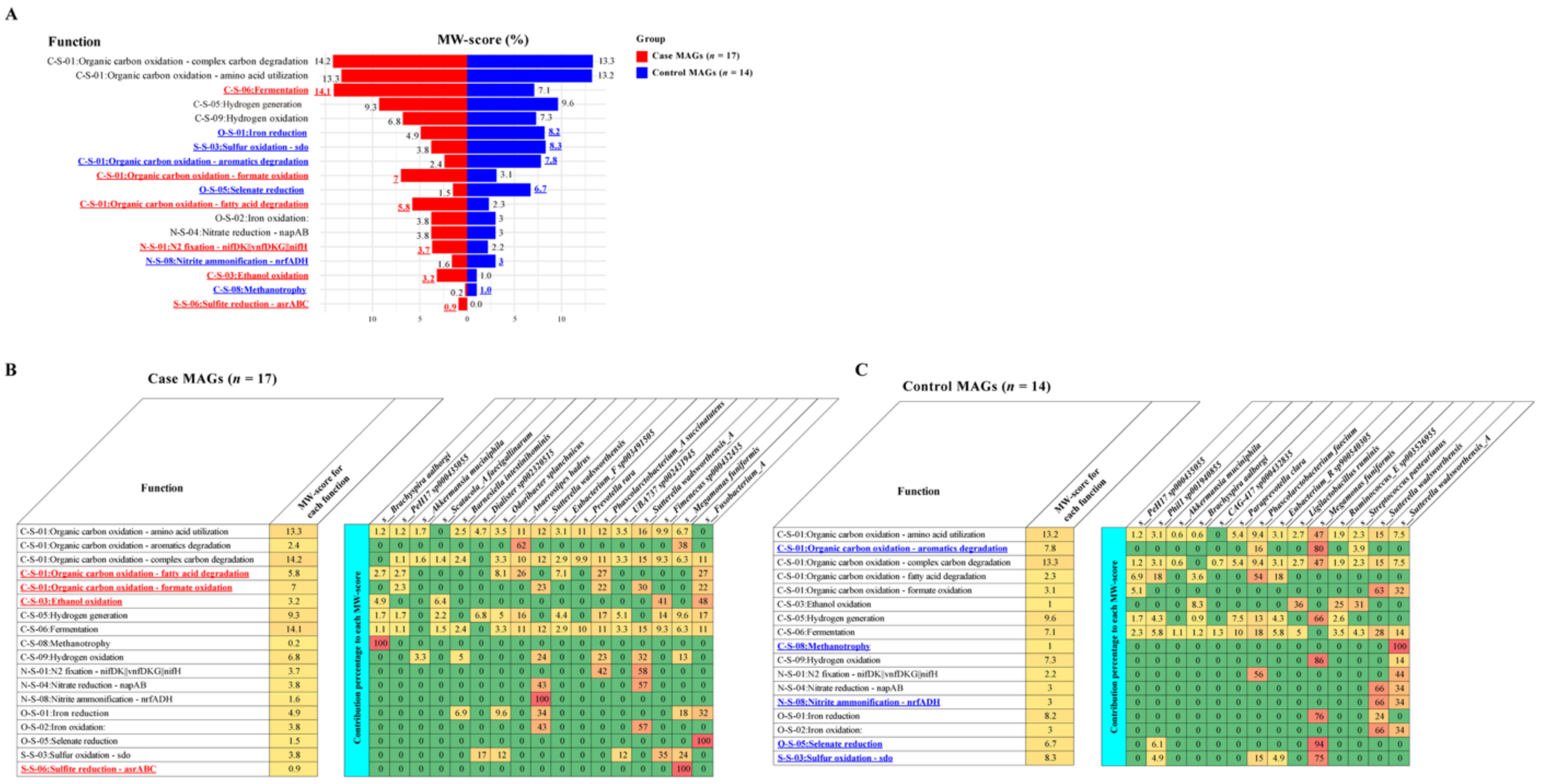

Fig. S6. Community-scale metabolic profiling of MAGs from co-assemblies in CAD and control groups using METABOLIC (v4.0). (A) Comparison of metabolic weight scores (MW-scores) between MAGs derived from the CAD (*n* = 17) and control (*n* = 14) groups. MW-scores represent the proportion of each metabolic function within the total functional capacity of the microbial community. Heatmaps showing species-level contributions to each metabolic function in CAD-derived MAGs **(B)** and control-derived MAGs **(C)**, calculated using the METABOLIC-C.pl function.

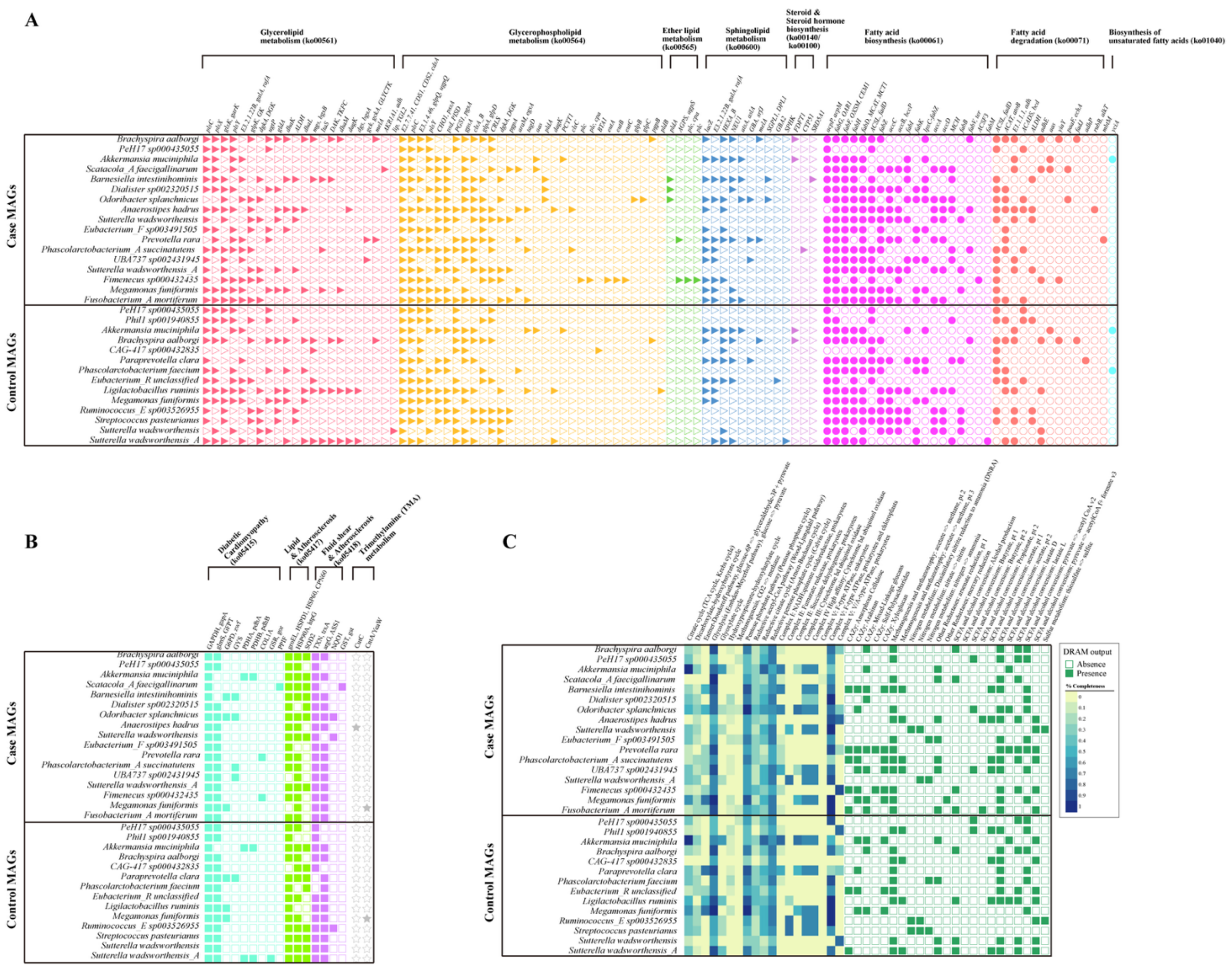

Fig. S7. Functional gene annotation of 31 MAGs identified from CAD cases and controls in this study: (A) with lipid metabolism (KEGG: 09103), (B) cardiovascular disease (KEGG: 09166) and TMA metabolism, and (C) DRAM-implemented functional modules. Visualization was performed using the iTol web server (v7, https://itol.embl.de/). CAD, coronary artery disease; MAG, metagenome-assembled genome.

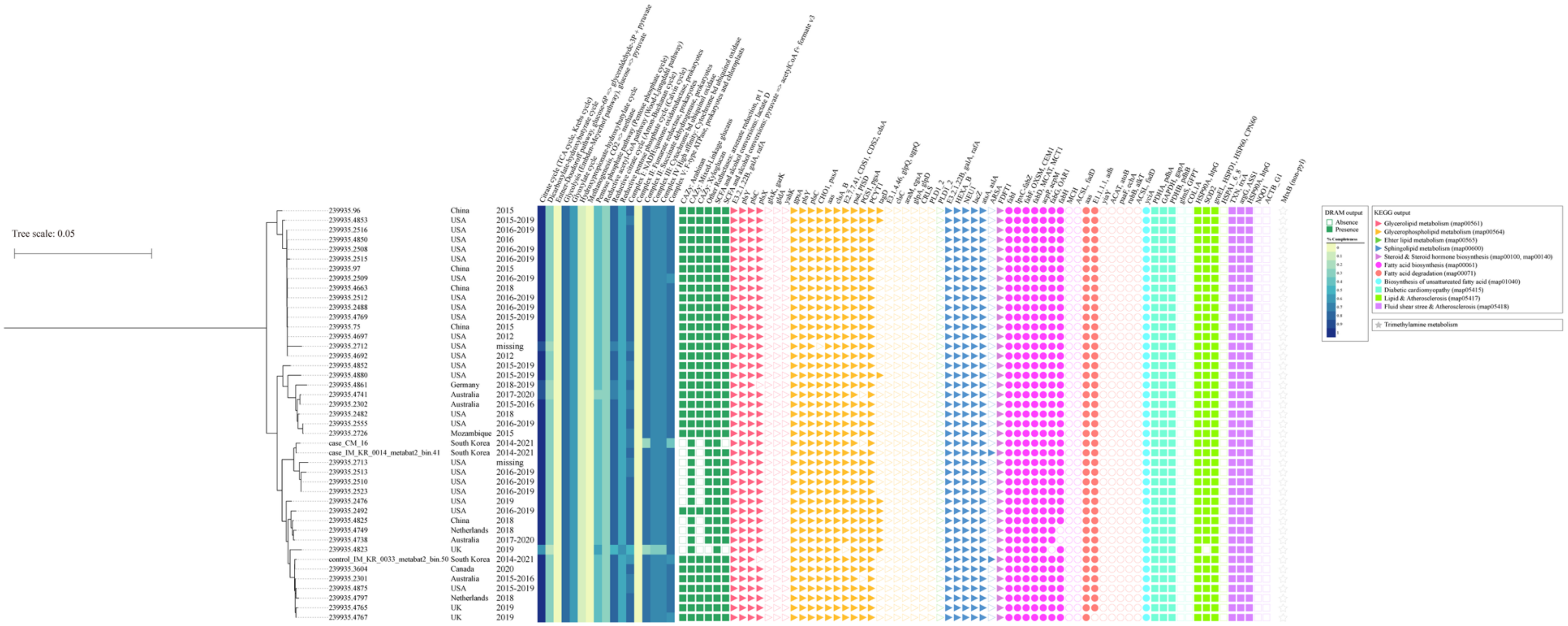

Tree scale: 0.05
239935.96 China 2015
239935.4853 USA 2015-2019
239935.2516 USA 2016-2019
239935.4850 USA 2016
239935.2508 USA 2016-2019
239935.2515 USA 2016-2019
239935.97 China 2015
239935.2509 USA 2016-2019
239935.4663 China 2018
239935.2512 USA 2016-2019
239935.2488 USA 2016-2019
239935.4769 USA 2015-2019
239935.75 China 2015
239935.4697 USA 2012
239935.2712 USA missing
239935.4692 USA 2012
239935.4852 USA 2015-2019
239935.4880 USA 2015-2019
239935.4861 Germany 2018-2019
239935.4741 Australia 2017-2020
239935.2302 Australia 2015-2016
239935.2482 USA 2018
239935.2555 USA 2016-2019
239935.2726 Mozambique 2015
case_CM_16 South Korea 2014-2021
case_IM_KR_0014_metabat2_bin.41 South Korea 2014-2021
239935.2713 USA missing
239935.2513 USA 2016-2019
239935.2510 USA 2016-2019
239935.2523 USA 2016-2019
239935.2476 USA 2019
239935.2492 USA 2016-2019
239935.4825 China 2018
239935.4749 Netherlands 2018
239935.4738 Australia 2017-2020
239935.4823 UK 2019
control_IM_KR_0033_metabat2_bin.50 South Korea 2014-2021
239935.3604 Canada 2020
239935.2301 Australia 2015-2016
239935.4875 USA 2015-2019
239935.4797 Netherlands 2018
239935.4765 UK 2019
239935.4767 UK 2019
MtxB (non-pyl)
DRAM output
Absence
Presence
% Completeness
0
0.1
0.2
0.3
0.4
0.5
0.6
0.7
0.8
0.9
1
KEGG output
Glycerolipid metabolism (map00561)
Glycerophospholipid metabolism (map00564)
Ehter lipid metabolism (map00565)
Sphingolipid metabolism (map00600)
Steroid & Steroid hormone biosynthesis (map00100, map00140)
Fatty acid biosynthesis (map00061)
Fatty acid degradation (map00071)
Biosynthesis of unsattureated fatty acid (map01040)
Diabetic cardiomyopathy (map05415)
Lipid & Atherosclerosis (map05417)
Fluid shear stree & Atherosclerosis (map05418)
Trimethylamine metabolism

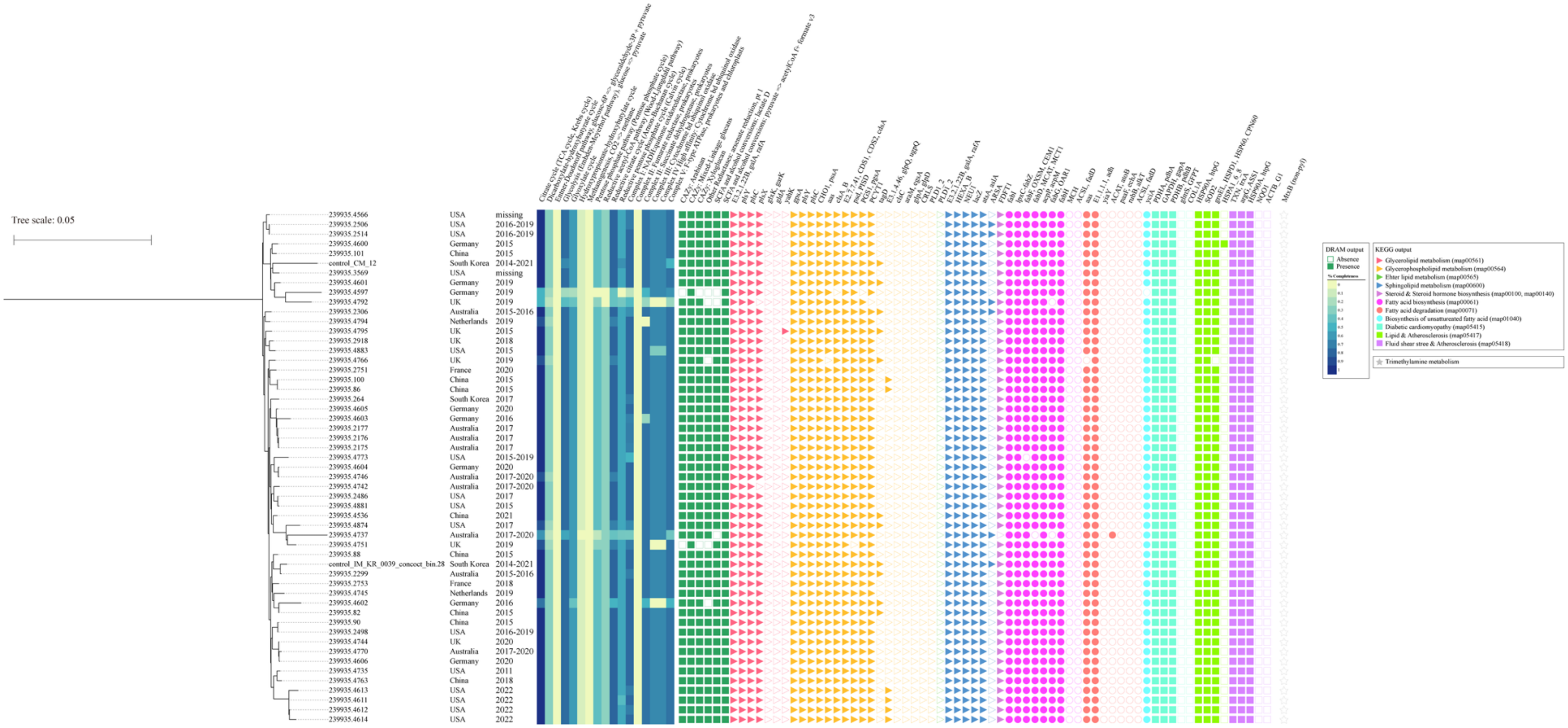

Fig. S8. Functional gene annotation of *Akkermansia muciniphila* strains identified from this study as well as whole genome sequence uploaded in the public database Bacterial and Viral Bioinformatics Resource Center (BV-BRC) with lipid metabolism (KEGG: 09103), cardiovascular disease (KEGG:09166), TMA metabolism, and DRAM-implemented functional modules. Based on phylogenetic distance (tree scale < 0.1), a total of 81 strains were selected from 429 reference strains. Phylogenetic trees were constructed with PhyloPhlAn (v.3.0). Visualization was performed using the iTol web server (v7, https://itol.embl.de/). Abbreviation. CAD, coronary artery disease; MAG, metagenome-assembled genome; CM, co-assembly MAG; IM, individual-assembly MAG.

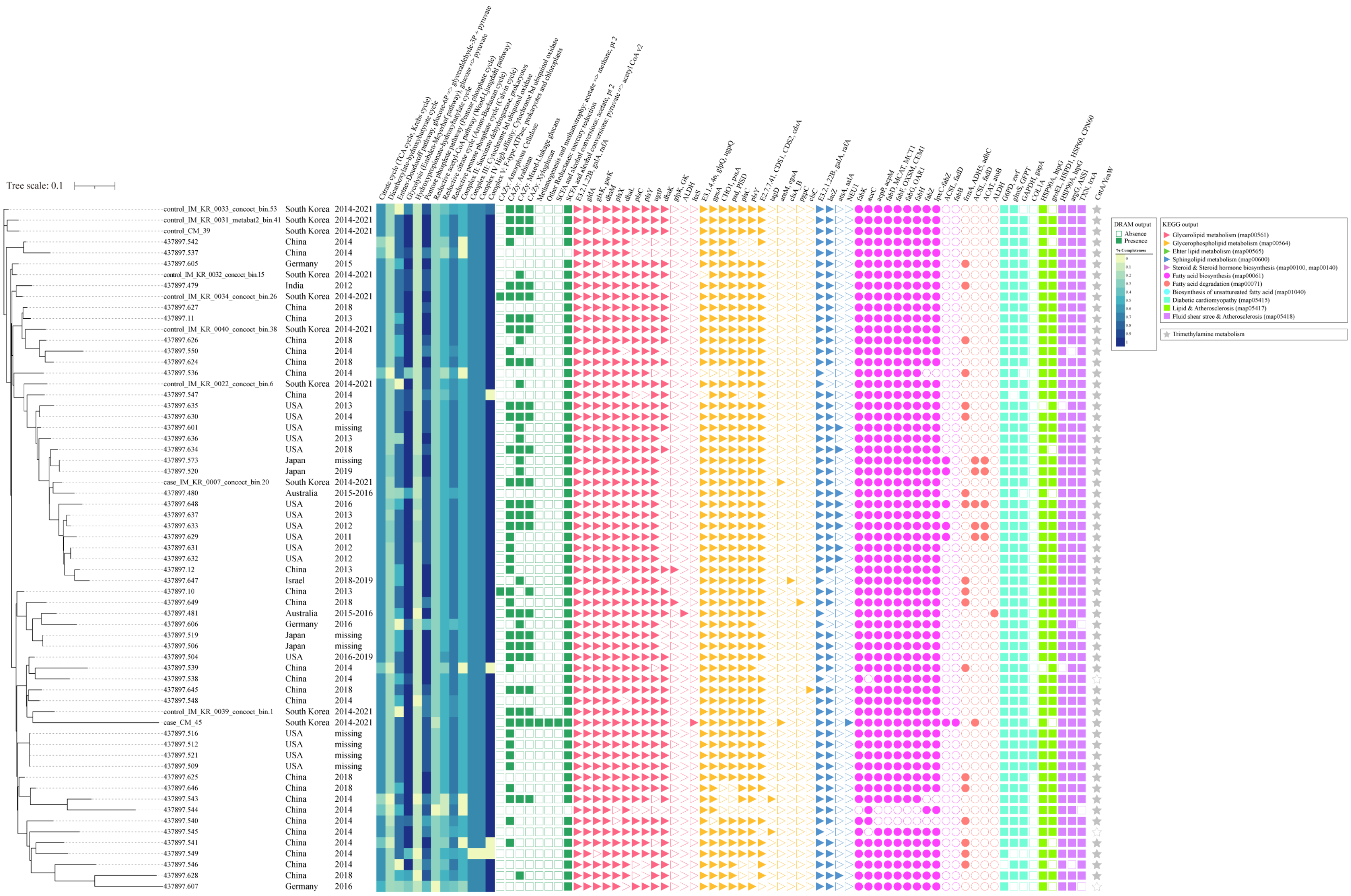

Fig. S9. Functional gene annotation of *Megamonas fumiformis* strains identified from this study as well as whole genome sequence uploaded in the public database BV-BRC with lipid metabolism (KEGG: 09103), cardiovascular disease (KEGG: 09166), TMA

metabolism, and DRAM-implemented functional modules. Phylogenetic trees were constructed with PhyloPhlAn (v.3.0). Visualization was performed using the iTol web server (v7, https://itol.embl.de/). Abbreviation. CAD, coronary artery disease; MAG, metagenome-assembled genome; CM, co-assembly MAG; IM, individual-assembly MAG.

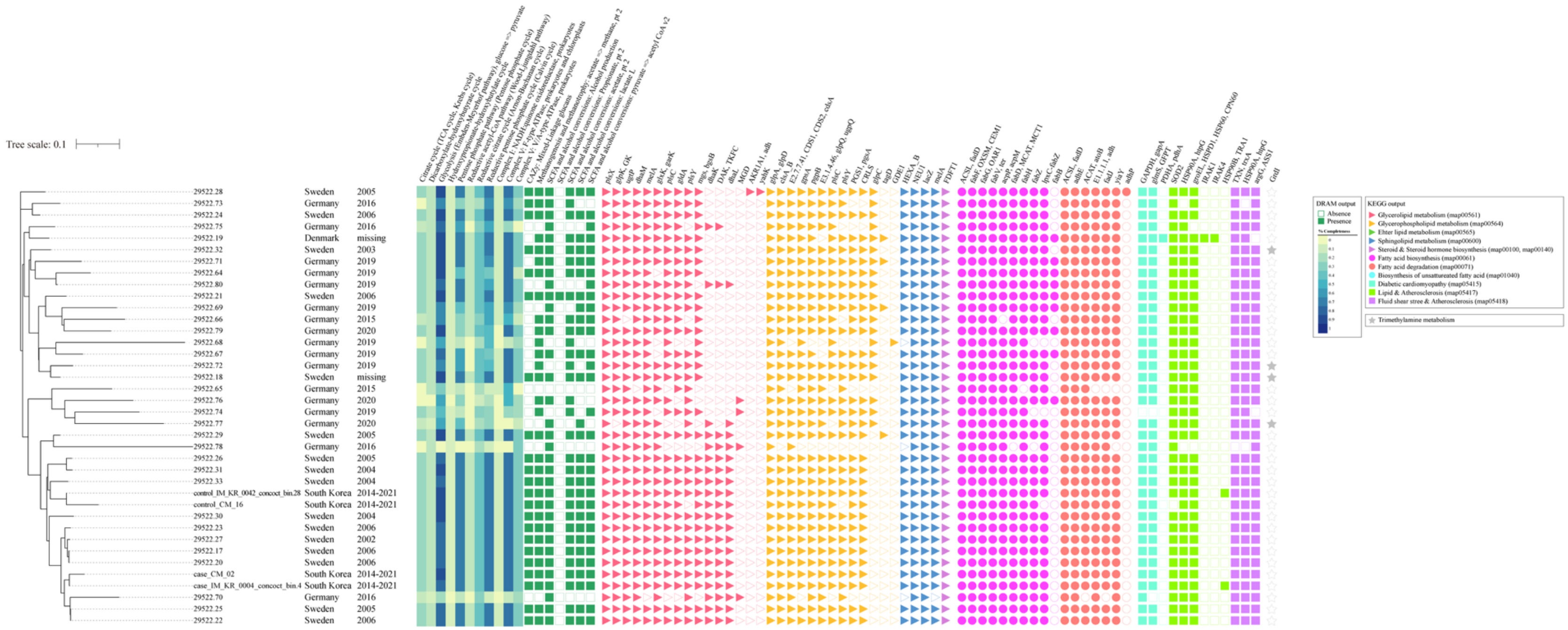

Fig. S10. Functional gene annotation of *Brachyspira aalborgi* strains identified from this study as well as whole genome sequence uploaded in the public database BV-BRC with lipid metabolism (KEGG: 09103), cardiovascular disease (KEGG: 09166), TMA metabolism, and DRAM-implemented functional modules. Phylogenetic trees were constructed with PhyloPhlAn (v.3.0). Visualization was performed using the iTol web server (v7, https://itol.embl.de/). Abbreviation. CAD, coronary artery disease; MAG, metagenome-assembled genome; CM, co-assembly MAG; IM, individual-assembly MAG.

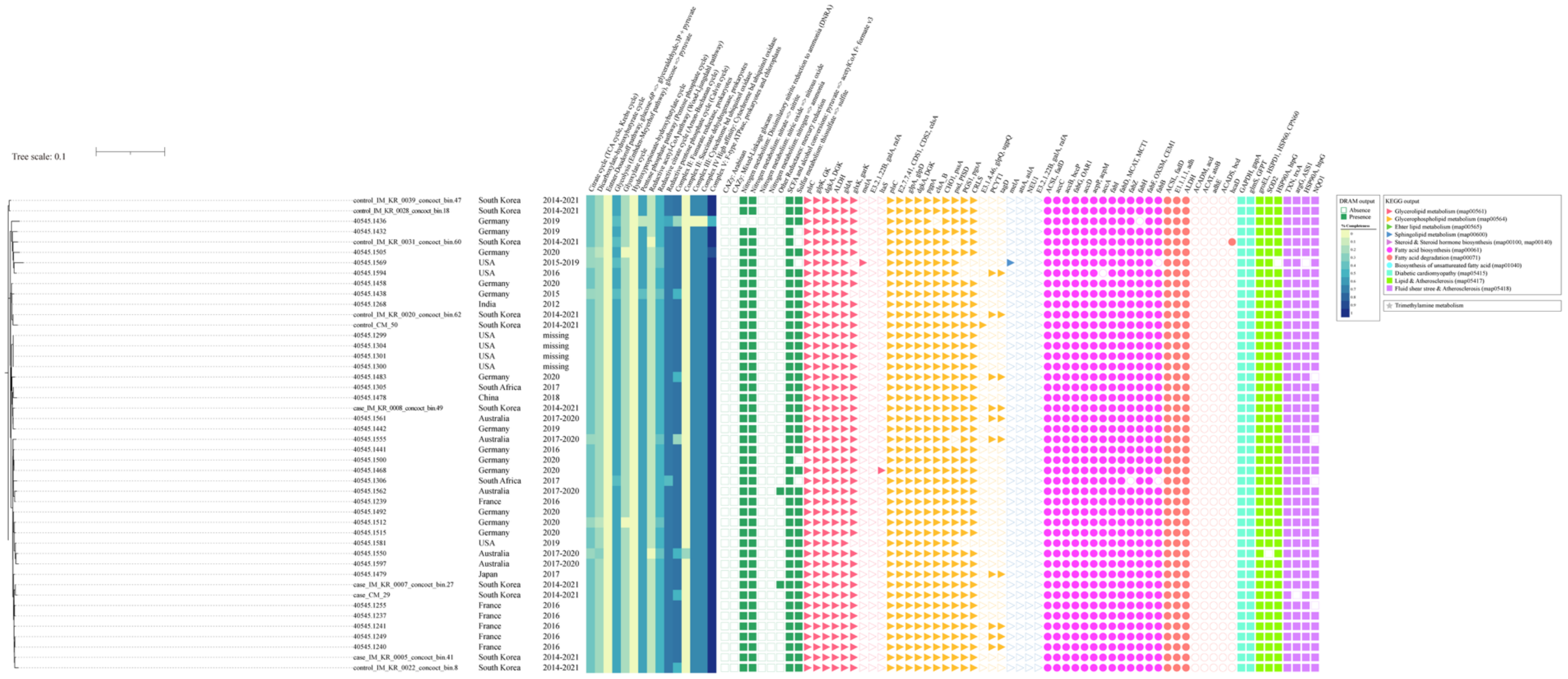
Tree scale: 0.1
control_IM_KR_0039_concoct_bin.47 South Korea 2014-2021
control_IM_KR_0028_concoct_bin.18 South Korea 2014-2021
40545.1436 Germany 2019
40545.1432 Germany 2019
control_IM_KR_0031_concoct_bin.60 South Korea 2014-2021
40545.1505 Germany 2020
40545.1569 USA 2015-2019
40545.1594 USA 2016
40545.1458 Germany 2020
40545.1438 Germany 2015
40545.1268 India 2012
control_IM_KR_0020_concoct_bin.62 South Korea 2014-2021
control_CM_50 South Korea 2014-2021
40545.1299 USA missing
40545.1304 USA missing
40545.1301 USA missing
40545.1300 USA missing
40545.1483 Germany 2020
40545.1305 South Africa 2017
40545.1478 China 2018
case_IM_KR_0008_concoct_bin.49 South Korea 2014-2021
40545.1561 Australia 2017-2020
40545.1442 Germany 2019
40545.1555 Australia 2017-2020
40545.1441 Germany 2016
40545.1500 Germany 2020
40545.1468 Germany 2020
40545.1306 South Africa 2017
40545.1562 Australia 2017-2020
40545.1239 France 2016
40545.1492 Germany 2020
40545.1512 Germany 2020
40545.1515 Germany 2020
40545.1581 USA 2019
40545.1550 Australia 2017-2020
40545.1597 Australia 2017-2020
40545.1479 Japan 2017
case_IM_KR_0007_concoct_bin.27 South Korea 2014-2021
case_CM_29 South Korea 2014-2021
40545.1255 France 2016
40545.1237 France 2016
40545.1241 France 2016
40545.1249 France 2016
40545.1240 France 2016
case_IM_KR_0005_concoct_bin.41 South Korea 2014-2021
control_IM_KR_0022_concoct_bin.8 South Korea 2014-2021
DRAM output
Absence
Presence
% Completeness
0
0.1
0.2
0.3
0.4
0.5
0.6
0.7
0.8
0.9
1
KEGG output
Glycerolipid metabolism (map00561)
Glycerophospholipid metabolism (map00564)
Ehter lipid metabolism (map00565)
Sphingolipid metabolism (map00600)
Steroid & Steroid hormone biosynthesis (map00100, map00140)
Fatty acid biosynthesis (map00061)
Fatty acid degradation (map00071)
Biosynthesis of unsaturated fatty acid (map01040)
Diabetic cardiomyopathy (map05415)
Lipid & Atherosclerosis (map05417)
Fluid shear stree & Atherosclerosis (map05418)
Trimethylamine metabolism

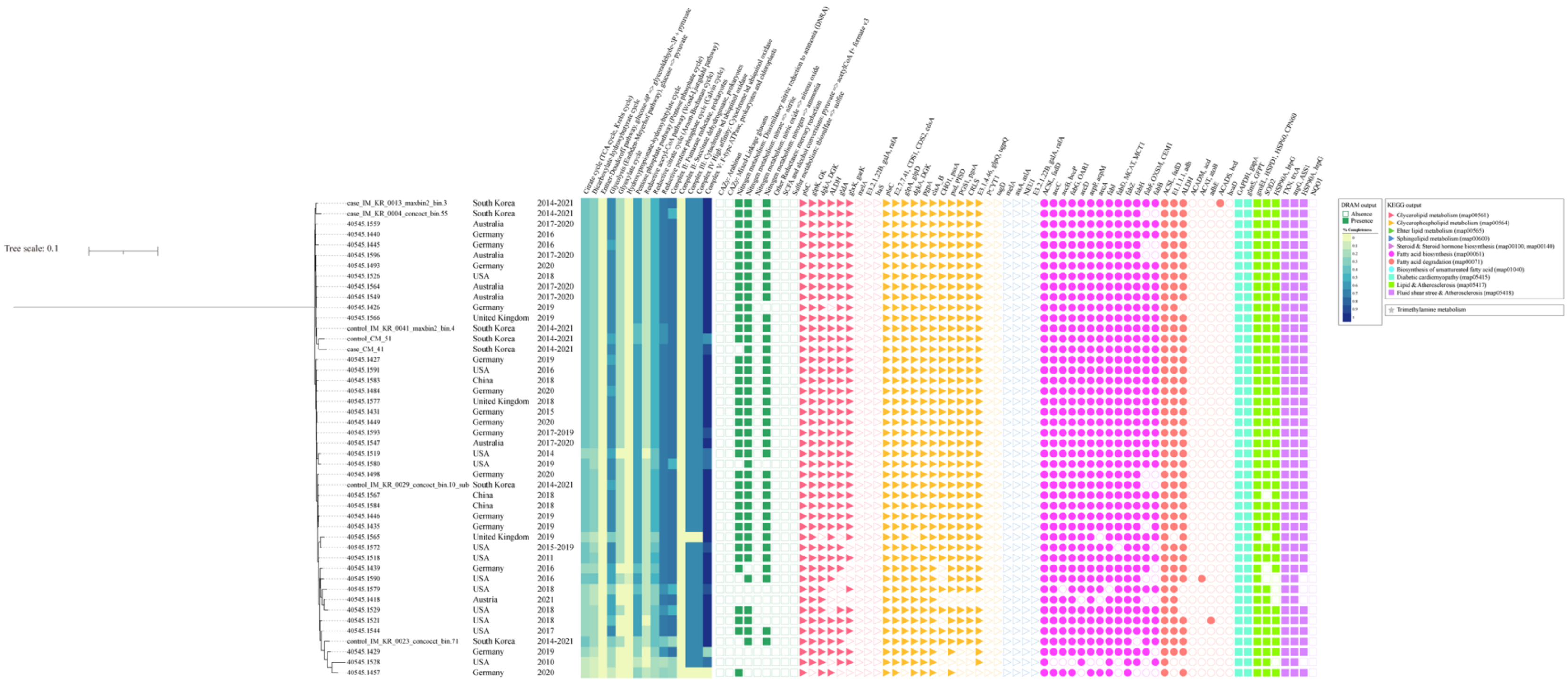

Fig. S11. Functional gene annotation of *Sutterella wadsworthensis* strains identified from this study as well as whole genome sequence uploaded in the public database BV-BRC with lipid metabolism (KEGG: 09103), cardiovascular disease (KEGG: 09166), TMA metabolism, and DRAM-implemented functional modules. From a total of 204 reference strains, 75 strains were selected based on phylogenetic distance (tree scale < 0.1). Phylogenetic trees were constructed with PhyloPhlAn (v.3.0). Visualization was performed using the iTol web server (v7, https://itol.embl.de/). Abbreviation. CAD, coronary artery disease; MAG, metagenome-assembled genome; CM, co-assembly MAG; IM, individual-assembly MAG.

References

1. Chang Y, Cho YK, Kim Y, Sung E, Ahn J, Jung HS, Yun KE, Shin H, Ryu S. 2019. Nonheavy Drinking and Worsening of Noninvasive Fibrosis Markers in Nonalcoholic Fatty Liver Disease: A Cohort Study. Hepatology 69:64-75.
2. Bolger AM, Lohse M, Usadel B. 2014. Trimmomatic: a flexible trimmer for Illumina sequence data. Bioinformatics 30:2114-20.
3. Li H. 2013. Aligning sequence reads, clone sequences and assembly contigs with BWA-MEM. arXiv preprint arXiv:13033997.
4. Li H, Handsaker B, Wysoker A, Fennell T, Ruan J, Homer N, Marth G, Abecasis G, Durbin R, Genome Project Data Processing S. 2009. The Sequence Alignment/Map format and SAMtools. Bioinformatics 25:2078-9.
